# Comprehensive bioinformatics analysis and systems biology approaches to identify the interplay between COVID-19 and pericarditis

**DOI:** 10.3389/fimmu.2024.1264856

**Published:** 2024-02-22

**Authors:** Daisong Li, Ruolan Chen, Chao Huang, Guoliang Zhang, Zhaoqing Li, Xiaojian Xu, Banghui Wang, Bing Li, Xian-Ming Chu

**Affiliations:** ^1^ Department of Cardiology, The Affiliated Hospital of Qingdao University, Qingdao, China; ^2^ Department of Genetics and Cell Biology, Basic Medical College, Qingdao University, Qingdao, China; ^3^ Department of Dermatology, The Affiliated Haici Hospital of Qingdao University, Qingdao, China; ^4^ Department of Cardiology, The Affiliated Cardiovascular Hospital of Qingdao University, Qingdao, China

**Keywords:** COVID-19, pericarditis, common genes, bioinformatics analysis, immunology

## Abstract

**Background:**

Increasing evidence indicating that coronavirus disease 2019 (COVID-19) increased the incidence and related risks of pericarditis and whether COVID-19 vaccine is related to pericarditis has triggered research and discussion. However, mechanisms behind the link between COVID-19 and pericarditis are still unknown. The objective of this study was to further elucidate the molecular mechanisms of COVID-19 with pericarditis at the gene level using bioinformatics analysis.

**Methods:**

Genes associated with COVID-19 and pericarditis were collected from databases using limited screening criteria and intersected to identify the common genes of COVID-19 and pericarditis. Subsequently, gene ontology, pathway enrichment, protein–protein interaction, and immune infiltration analyses were conducted. Finally, TF–gene, gene–miRNA, gene–disease, protein–chemical, and protein–drug interaction networks were constructed based on hub gene identification.

**Results:**

A total of 313 common genes were selected, and enrichment analyses were performed to determine their biological functions and signaling pathways. Eight hub genes (*IL-1β*, *CD8A*, *IL-10*, *CD4*, *IL-6*, *TLR4*, *CCL2*, and *PTPRC*) were identified using the protein–protein interaction network, and immune infiltration analysis was then carried out to examine the functional relationship between the eight hub genes and immune cells as well as changes in immune cells in disease. Transcription factors, miRNAs, diseases, chemicals, and drugs with high correlation with hub genes were predicted using bioinformatics analysis.

**Conclusions:**

This study revealed a common gene interaction network between COVID-19 and pericarditis. The screened functional pathways, hub genes, potential compounds, and drugs provided new insights for further research on COVID-19 associated with pericarditis.

## 1 Introduction

COVID-19 is an atypical respiratory disease caused by the severe acute respiratory syndrome coronavirus 2 (SARS-CoV-2), which has triggered a global pandemic and caused significant loss of life and property (1). Globally, as of 21 January, there were 774,395,593 confirmed cases of COVID-19, including 7,023,271 deaths, reported by the World Health Organization (WHO) (https://covid19.who.int/). Although more than 80% of patients with COVID-19 present with asymptomatic infection or mild to moderate self-resolving symptoms, more than 15% of patients still develop into severe cases, manifested as severe pneumonia or acute respiratory distress syndrome, and even multiple organ failure (2, 3). In addition to respiratory diseases, cardiovascular complications have gradually become a major threat for patients with COVID-19. Pericarditis is the most common pericardial disease worldwide; the pericardium provides fixation and physical protection for the heart, such as slowing down the impact of heart contraction on the surrounding blood vessels and preventing the spread of pulmonary and thoracic infections (4). The etiology of pericarditis may be infectious (bacterial or viral) or noninfectious (systemic inflammatory disease or post-cardiac injury syndrome) (5). Viral infection is an important cause of pericarditis and studies have shown that it can be an early complication of COVID-19. Notably, the incidence rate of pericarditis has increased by at least 15 times after SARS-CoV-2 infection than before COVID-19, and estimates of excess cases associated with vaccination also indicate a burden associated with pericarditis (6, 7). Over 13 billion doses of COVID-19 vaccines have been administered; furthermore, several passive surveillance systems have indicated that the risk of pericarditis increased after COVID-19 vaccination, especially in young men, but authoritative research claimed that the incidence was rare (8). Considering benefits and risks, vaccination should be firmly supported, but strengthening the surveillance of adverse events following vaccination and continuing to study the mechanistic relationship between COVID-19 and pericarditis are still essential.

Increasing evidence suggests that immune responses and potential immune markers may be associated with COVID-19 severity. Differences in innate immune system components lead to heterogeneity in the COVID-19 disease spectrum (9). An imbalanced immune response during viral invasion is an important immunopathological mechanism in severe diseases (10). After SARS-CoV-2 infection, immune effector cells release a large number of pro-inflammatory cytokines, triggering a cytokine storm that causes important immunopathological events, such as ARDS and multiple organ failure (11). In recent years, immune checkpoints have led to breakthroughs and progress in cancer treatment; however, their application is still limited due to immune-related adverse events, such as cardiotoxicity. The onset and progression of pericarditis in the cardiotoxicity brought on by immunotherapy are intimately tied to the unrestricted regulation of the immune system (12). The predisposing factors and pathogenesis of pericarditis remain unclear, and may be related to viral infections or autoimmune-inflammatory diseases. Under the influence of exogenous triggers, infections may lead to an autoimmune response in susceptible hosts by activating innate immunity (13, 14). Therefore, gaining a comprehensive and in-depth understanding of interactions between viruses and the human immune system is necessary. Furthermore, studying the impact and mechanism of the clinical outcomes of COVID-19 and pericarditis is crucial to promote the research and development of vaccines with reduced side effects.

In recent years, with the rapid development of high-throughput biotechnology, the use of gene interaction networks in bioinformatics research has become increasingly convenient. The construction of gene interaction network not only helps to further understand various biological processes from the perspective of systems, but also can be widely applied to explore the pathogenesis of diseases. However, massive data cannot be verified one by one to explain the mechanism, so gene enrichment studies are needed to classify differential genes, so as to filter redundant data and screen out more valuable functional information (15). Based on data sources and algorithms, methods for gene functional enrichment analysis can be roughly divided into four categories: over-representative analysis (ORA), function set scoring (FCS), pathway topology (PT), and network topology (NT). Enrichment analyses commonly used include GO enrichment analysis, KEGG enrichment analysis and gene set enrichment analysis (GSEA). GSEA consists of three key elements: calculating enrichment scores, evaluating significance, and adjusting for multiple hypothesis tests. Weighted Kolmogorov Smirnov (WKS) test was used in GSEA to obtain the statistical value of the functional set of the tested gene, and there are other statistical algorithms, such as χ2-test, Mean test, Median test, Wilcoxon rank sum test, etc. (16, 17). The schematic overview for GSEA can be found in **Supplementary Figure 1** (https://www.gsea-msigdb.org/gsea/) (16).

**Figure 1:**
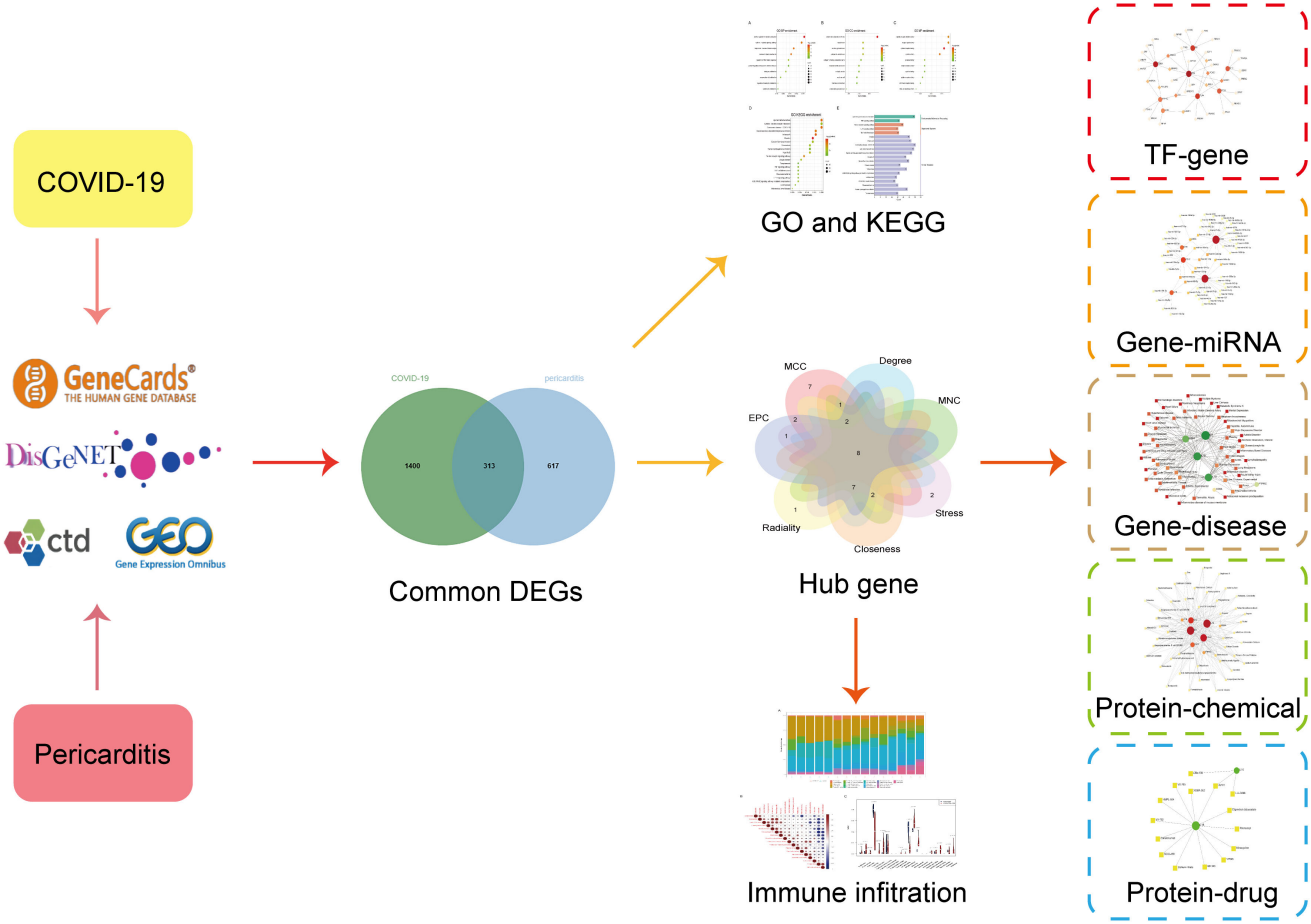
Workflow diagram of the study.

In this study, we employed a wide range of bioinformatics techniques to identify the common genes of COVID-19 and pericarditis and analyzed their enrichment pathways and functions. Protein–protein interaction (PPI) networks were constructed to identify hub genes and further analyze the interaction networks of transcription factors (TFs), microRNAs (miRNAs), chemicals, and drugs. The immune response can serve as a resection point for studying the common pathogenesis of comorbidities. To uncover molecular regulatory networks and investigate the relationship between hub genes and immune cells, immune infiltration analysis was employed. This study provides new insights for exploring the pathophysiological connections and immune mechanisms and excavating the potential biomarkers and therapeutic targets for COVID-19 and pericarditis. The overall flowchart of the study is shown in **Figure 1**.

## 2 Materials and methods

### 2.1 Dataset preparation

By searching the DisGeNET (https://www.disgenet.org/) (18), comparative toxicogenomics database (CTD) (http://ctdbase.org/) (19) and GeneCards (https://www.genecards.org/) (20) databases, we identified genes related to pericarditis and COVID-19. We selected supplementary datasets from Gene Expression Omnibus (GEO) of the National Center for Biotechnology Information (https://www.ncbi.nlm.nih.gov/geo/) (21). GSE164805, platform number GPL26963, the whole genome transcriptome of peripheral blood mononuclear cells was analyzed on five healthy controls and ten COVID-19 patients (22).

### 2.2 Identification of the common genes of COVID-19 and pericarditis

Based on the scoring standards of the different databases, we collected the top 500 genes from the DisGeNET, CTD, and GeneCards databases when the number was greater than 500. Online GEO analysis tool GEO2R (www.ncbi.nlm.nih.gov/geo/geo2r/) was used to analyze sample data for differential gene expression (21). We utilized GEO2R to identify the differentially expressed genes (DEGs) with a false discovery rate (FDR) < 0.00001 and |log fold-change| > 1 for GSE164805. Subsequently, we integrated these two parts of genes related to COVID-19 and then took the intersection of COVID-19 and pericarditis to obtain common genes using an online Venn tool (http://jvenn.toulouse.inra.fr/app/example.html) (23).

### 2.3 GO and KEGG pathway enrichment analyses

In order to investigate the probable biological connection between COVID-19 and pericarditis, the clusterProfiler software (version 3.14.3) was used to conduct Gene Ontology (GO) and Kyoto Encyclopedia of Genes and Genomes (KEGG) enrichment analysis for the common genes (24). Biological processes (BP), cellular elements (CC), and molecular functions (MF) were all included in the GO analysis. The top 10 GO and top 20 in KEGG items with the lowest p-values were shown as bubble diagrams using an online platform (http://www.bioinformatics.com.cn) for data processing and visualization.

### 2.4 PPI network analysis and hub genes screening

Identifying unknown protein functional modules from PPI networks is crucial for understanding protein function and interpreting key data in cell biology. PPI network analysis is a promising strategy that can provide a deeper and more comprehensive insight into the relationships between various diseases from the standpoint of protein interactions (25, 26). The online analytical tool STRING (https://string-db.org/) was to study protein interactions, systematically collect and integrate physical regulatory interactions and functional relationships between proteins (27). We constructed a PPI network based on the score greater than 0.4 and analyzed and visualized the results using Cytoscape 3.9.1, which is an open-source project designed to integrate high-throughput data and molecular interaction networks into a unitive framework (28). CytoHubba (https://apps.cytoscape.org/apps/cytohubba), an important Cytoscape plugin for network topology analysis, uses 11 methods for studying key genes from different perspectives.

### 2.5 Immune infiltration analysis

Immune cells exhibit specific patterns of infiltration and residence. Studying the infiltration status can provide a better understanding of their role and mechanism in disease pathogenesis and can thus be applied to the discovery of new treatment strategies for many diseases (29). The CIBERSORT tool, based on the linear support vector regression, decomposes the expression matrix of subtypes of human immune cells for immune-immersion analysis (30). The proportion of immune cells in GSE164805 was calculated, along with the relevance between immune cells and hub genes, as well as each immune cell.

### 2.6 Identification of TFs and miRNAs

TFs are proteins to recognize special DNA sequences and are key cellular components forming complex regulatory systems to control gene expression (31). NetworkAnalyst (http://www.networkanalyst.ca) is to conduct complex meta-analyses for gene expression and is suitable for data processing and analysis in the context of PPI networks (32). The construction of the TF–genes was based on the JASPAR database (http://jaspar.genereg.net), which includes TF-binding profiles of multiple species from six taxonomic groups (33). MiRNAs regulate protein expression by binding to TF; research on the interaction network of TF-miRNAs was conducted using the RegNetwork database (http://www.regnetworkweb.org/) (34). In addition to studying the role of target genes and miRNAs with TF, we carried out topology analysis and construction of gene–miRNA networks based on miRTarBase v8.0 (https://miRTarBase.cuhk.edu.cn/) (35).

### 2.7 Analysis of gene–disease interaction networks

DisGeNET integrates and standardizes disease-related genes and variant data, covering the whole spectrum of human diseases as well as normal and abnormal features (36). Gene–disease network was established was to study diseases related to COVID-19 and pericarditis using the NetworkAnalyst platform.

### 2.8 Analysis of protein–chemical and protein–drug interaction networks

Constructing protein–chemical and protein–drug networks is conducive to predicting the target information of drugs and chemicals relevant to COVID-19 and pericarditis. In the NetworkAnalyst platform, the corresponding compounds and drugs were identified and obtained using the CTD and DrugBank database.

## 3 Results

### 3.1 Identification of common genes of COVID-19 and pericarditis

By searching the DisGeNET, CTD, and GeneCards databases, we identified genes related to COVID-19 and pericarditis. To improve the integration and standardization of the data, we summarized the top 500 genes in each database according to their scoring standards. If the original data were less than 500, all retrieved data were included. Using this rule, we obtained 51, 500, and 500 pericarditis-related genes from the DisGeNET, CTD, and GeneCards databases, respectively. Subsequently, 930 pericarditis-related genes were identified by merging and de-duplicating the results from the three databases.

**Figure 2:**
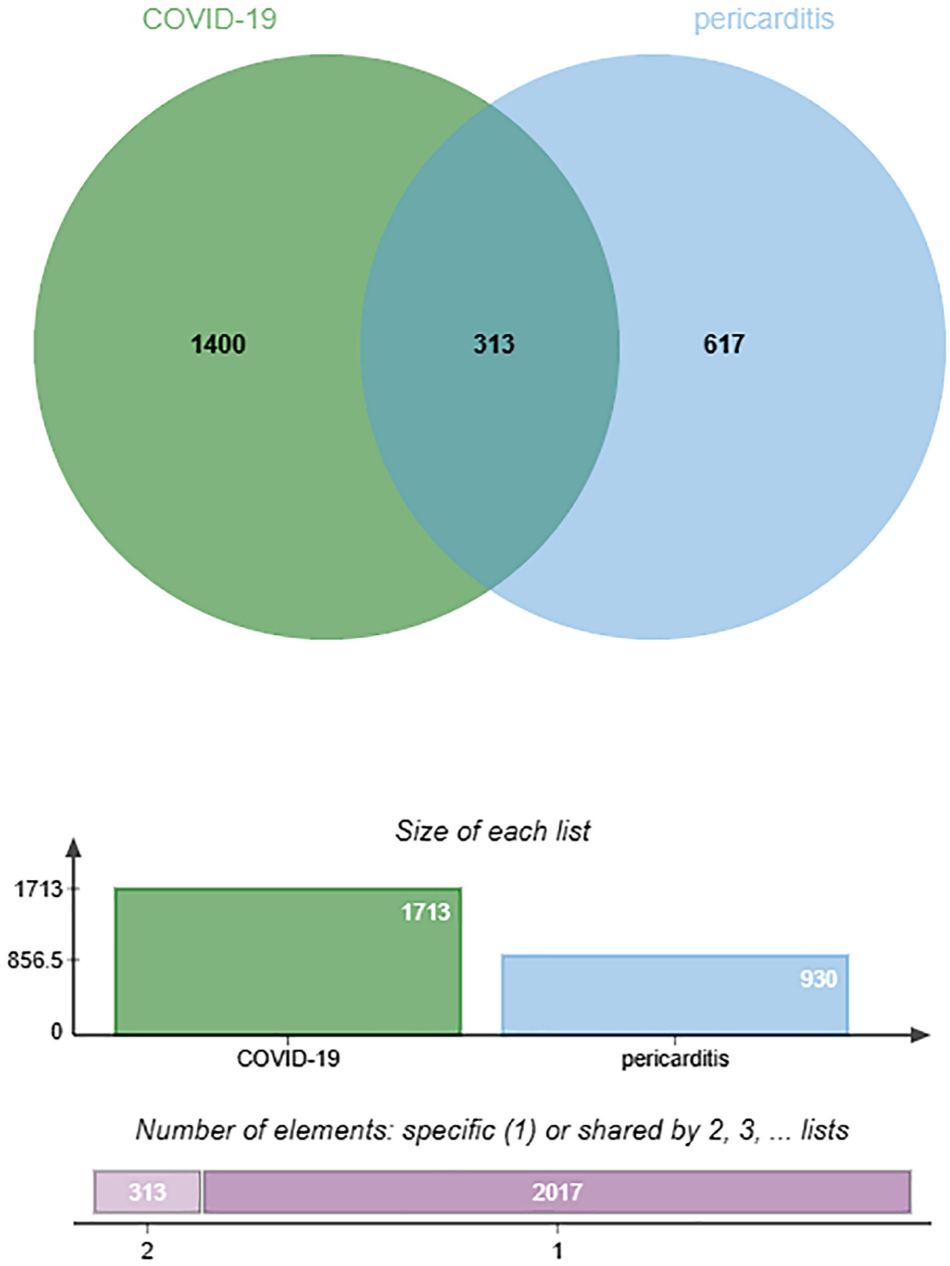
Common genes of COVID-19 and pericarditis displayed through Venn diagram.

Using the same method, we obtained 1236 COVID-19-related genes from DisGeNET, CTD, and GeneCards. In addition, we gained 494 COVID-related genes under the settings: FDR < 0.00001 and log fold-change > 1. By merging and deduplicating the results from the three databases and the GEO dataset, we obtained 1711 COVID-related genes. Finally, 313 common genes between COVID-19 and pericarditis were identified through intersections (**Figure 2**) (**Table 1**; **Supplementary Tables 1–3**).

**Table 1:** Collection of COVID-19 and pericarditis-related genes.

Disease	Data type	Data source	Raw number	Filter condition	After filtering	Merge	Common
pericarditis	Database	GeneCards	1008	If the raw data are less than 500, all are included	500	930	313
COVID-19	Database GEO	DisGeNET CTD GeneCards DisGeNET CTD GSE164805	51 29276 6051 1843 9896 8953	FDR < 0.00001 and log Fold-Change > 1	51 500 500 500 500 494	1711	

### 3.2 GO and KEGG pathway enrichment analyses

**Figure 3:**
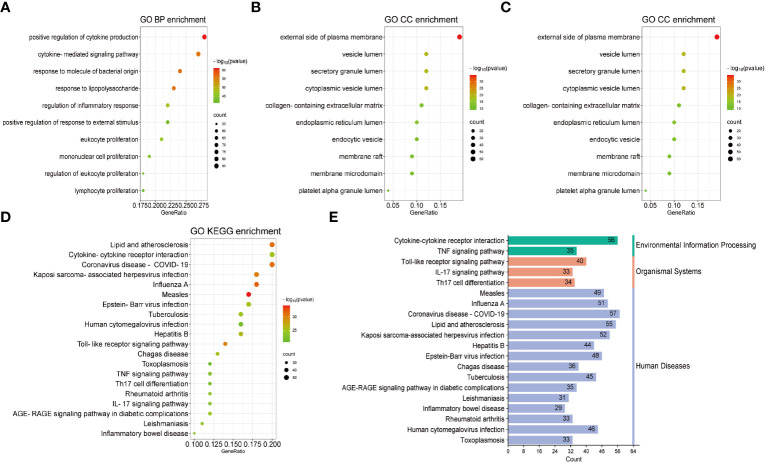
GO and KEGG enrichment analysis of the common genes. **(A)** Biological processes in bubble chart. **(B)** Cellular component in bubble chart. **(C)** Molecular function in bubble chart. **(D)** The top 20 signaling pathways of KEGG in bubble chart. **(E)** The top 20 signaling pathways of KEGG in bar graph. The color of the dots reflects the size of the p-values, and the size of the dots reflects the number of annotated genes in the bubble charts. Different colors represent different pathway classifications, and the horizontal axis represents the number of genes per pathway in a bar graph.

Based on common genes, GO and KEGG enrichment were carried out to examine their biological roles and signal pathways. **Figure 3** displayed the top 10 terms in the BP, CC, and MF categories with the lowest p-values. BP terms mainly involved the regulation of cytokine production and inflammatory response and were associated with the proliferation of immune cells, involving in lymphocytes, leukocytes, and mononuclear cells (**Figure 3A**). CC terms mainly revealed the external side of the plasma membrane and some lumens, such as secretory granules, cytoplasmic vesicles, and the endoplasmic reticulum (**Figure 3B**). MF terms mainly demonstrated the activity of signaling receptors and immune receptors which are crucial for the binding of various factors, including proteases, cytokines, and chemokines (**Figure 3C**). Furthermore, KEGG analysis showed that most pathways were involved in immune- and infection-related diseases, such as COVID-19, influenza, measles, tuberculosis, rheumatoid arthritis, hepatitis and inflammatory bowel disease. Notably, multiple immune-related pathways were also enriched, including cytokine receptor interaction, T helper 17 (Th17) cell differentiation, interleukin 17 (IL-17), tumor necrosis factor (TNF), and Toll-like receptor signaling pathways (**Figures 3D, E**). All results were visualized using bubble plots, which manifested that common genes might be involved in immune-related functions and pathways, thereby affecting the progression of COVID-19 and pericarditis (**Tables 2**, **3**).

**Table 2:** The top 10 items of GO enrichment of the common genes.

Ontology	ID	Description	GeneRatio	P value	Count
BP BP BP BP BP BP BP BP BP BP	GO:0001819 GO:0002237 GO:0032496 GO:0019221 GO:0050727 GO:0070661 GO:0032943 GO:0070663 GO:0032103 GO:0046651	positive regulation of cytokine production response to molecule of bacterial origin response to lipopolysaccharide cytokine-mediated signaling pathway regulation of inflammatory response leukocyte proliferation mononuclear cell proliferation regulation of leukocyte proliferation positive regulation of response to external stimulus lymphocyte proliferation	0.28 0.24 0.23 0.27 0.22 0.21 0.19 0.18 0.22 0.18	7.0409E-62 1.85963E-57 4.72483E-57 1.08708E-56 4.33984E-48 2.68703E-47 5.28806E-45 5.02574E-43 1.41146E-42 9.36141E-42	86 74 72 82 69 64 60 55 67 57
CC CC CC CC CC CC CC CC CC CC MF MF MF MF MF MF MF MF MF MF	GO:0009897 GO:0060205 GO:0031983 GO:0034774 GO:0005788 GO:0062023 GO:0030139 GO:0045121 GO:0098857 GO:0031093 GO:0005126 GO:0005125 GO:0048018 GO:0030546 GO:0002020 GO:0019955 GO:0004896 GO:0140375 GO:0042379 GO:0003953	external side of plasma membrane cytoplasmic vesicle lumen vesicle lumen secretory granule lumen endoplasmic reticulum lumen collagen-containing extracellular matrix endocytic vesicle membrane raft membrane microdomain platelet alpha granule lumen cytokine receptor binding cytokine activity receptor ligand activity signaling receptor activator activity protease binding cytokine binding cytokine receptor activity immune receptor activity chemokine receptor binding NAD+ nucleosidase activity	0.19 0.12 0.12 0.12 0.10 0.11 0.10 0.09 0.09 0.04 0.16 0.14 0.18 0.18 0.07 0.07 0.06 0.07 0.05 0.03	2.5535E-35 5.3354E-20 5.3354E-20 2.15233E-19 2.08021E-14 5.89812E-13 1.43902E-12 2.32352E-12 2.32352E-12 1.18011E-09 9.19346E-35 1.41623E-28 1.51612E-26 2.32268E-26 8.36225E-15 2.25206E-12 2.77929E-12 4.54612E-12 6.76235E-10 8.86876E-10	60 38 38 37 31 34 30 29 29 13 50 42 54 54 23 21 18 21 14 10

**Table 3:** The top 20 items of KEGG enrichment of the common genes.

Ontology	ID	Description	GeneRatio	P value	Count
KEGG KEGG KEGG KEGG KEGG KEGG KEGG KEGG KEGG KEGG KEGG KEGG KEGG KEGG KEGG KEGG KEGG KEGG KEGG KEGG	hsa05162 hsa05164 hsa05171 hsa05417 hsa05167 hsa04620 hsa05161 hsa05169 hsa05142 hsa05152 hsa04060 hsa04933 hsa05140 hsa05321 hsa05323 hsa04657 hsa04668 hsa04659 hsa05163 hsa05145	Measles Influenza A Coronavirus disease - COVID-19 Lipid and atherosclerosis Kaposi sarcoma-associated herpesvirus infection Toll-like receptor signaling pathway Hepatitis B Epstein-Barr virus infection Chagas disease Tuberculosis Cytokine-cytokine receptor interaction AGE-RAGE signaling pathway in diabetic complications Leishmaniasis Inflammatory bowel disease Rheumatoid arthritis IL-17 signaling pathway TNF signaling pathway Th17 cell differentiation Human cytomegalovirus infection Toxoplasmosis	0.17 0.18 0.20 0.20 0.18 0.14 0.16 0.17 0.13 0.16 0.20 0.12 0.11 0.10 0.12 0.12 0.12 0.12 0.16 0.12	6.93254E-35 1.38095E-32 8.10128E-32 9.73817E-32 4.33964E-31 1.34666E-30 1.85229E-26 3.642E-26 4.80157E-26 1.47638E-25 1.49235E-25 2.79959E-25 1.48981E-24 1.51245E-24 4.03091E-24 5.6493E-24 1.65333E-23 5.736E-23 2.07707E-22 2.69548E-21	49 51 57 55 52 40 44 48 36 45 56 35 31 29 33 33 35 34 46 33

### 3.3 PPI network and hub gene analyses

**Figure 4:**
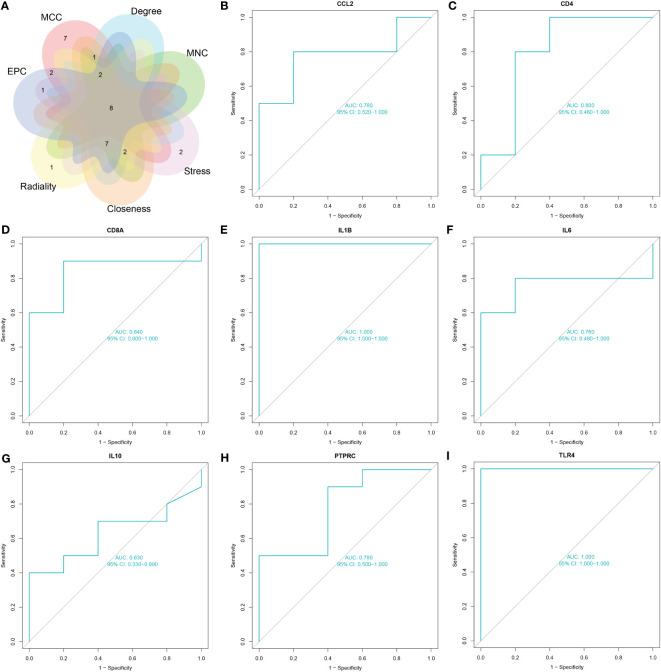
Screening and validation of hub genes. **(A)** The Venn diagram shows eight overlapping hub genes screened via the six algorithms. **(B–I)** Results of the ROC curve analysis and AUC values of hub genes in the COVID-19 dataset.

The common genes of COVID-19 and pericarditis were imported to STRING to create a PPI network, and then were uploaded into Cytoscape for comprehensive analysis to forecast gene interactions and associated pathways. CytoHubba is a plugin that identifies hub nodes and provides 11 analysis algorithms to calculate and sort nodes in the network. We use seven algorithms to calculate the top 20 and then take intersections to screen hub genes: Maximal Clique Centrality (MCC), Maximum Neighborhood Component (MNC), Degree, Closeness, Radiality, Stress and Edge Percolated Component (EPC) (**Figure 4A**) (**Table 4**). Interleukin 1 beta (*IL-1β*), cluster of differentiation 8 antigen (CD8A), interleukin 10 (*IL-10*), cluster of differentiation 4 (*CD4*), interleukin 6 (*IL-6*), Toll-like receptor 4 (*TLR4*), chemokine ligand 2 (*CCL2*), and Protein Tyrosine Phosphatase Receptor Type C (*PTPRC*) were among the top 20 genes identified from the seven algorithm scores. The area under the curve (AUC) results were to assess the specificity and sensitivity of the eight hub genes to COVID-19 using receiver operating characteristic (ROC) curve analysis. The AUC values of the hub genes were greater than 0.75, except for *IL-10*, indicating that these genes may be potential biomarkers and have a high diagnostic value for disease (**Figures 4B–I**).

**Table 4:** Top 20 hub genes in seven algorithms.

MCC	Degree	MNC	Stress	Closeness	Radiality	EPC
IFNG IL1B ITGAM CD8A CSF2 IL2 IL4 IL10 CD4 IL17A IL6 TLR4 CCL2 CXCL10 IL18 PTPRC IL13 CCL5 TLR2 CCL3	TNF IL6 IL1B ALB AKT1 CD4 IL10 CCL2 STAT3 VEGFA CXCL8 TLR4 INS TP53 MMP9 CD8A IFNG PTPRC IL4 ITGAM	TNF IL6 IL1B ALB AKT1 CD4 IL10 CCL2 STAT3 VEGFA CXCL8 TLR4 INS MMP9 TP53 CD8A IFNG PTPRC IL4 ITGAM	ALB TNF TP53 AKT1 IL6 IL1B CD4 INS STAT3 IL10 VEGFA CCL2 TLR4 CXCL8 CASP3 PTPRC MMP9 STAT1 CD8A ERBB2	TNF IL6 IL1B ALB AKT1 CD4 IL10 CCL2 STAT3 VEGFA CXCL8 TLR4 INS TP53 MMP9 CD8A IFNG PTPRC IL4 ITGAM	TNF IL6 IL1B ALB AKT1 CD4 IL10 CCL2 STAT3 VEGFA CXCL8 TLR4 INS MMP9 TP53 CD8A IFNG IL4 PTPRC ICAM1	TNF IL6 CD4 IL1B AKT1 CCL2 IFNG STAT3 TLR4 IL10 ALB IL4 CXCL8 MMP9 IL2 PTPRC CD8A IL13 VEGFA STAT1

### 3.4 Immune infiltration analysis

**Figure 5:**
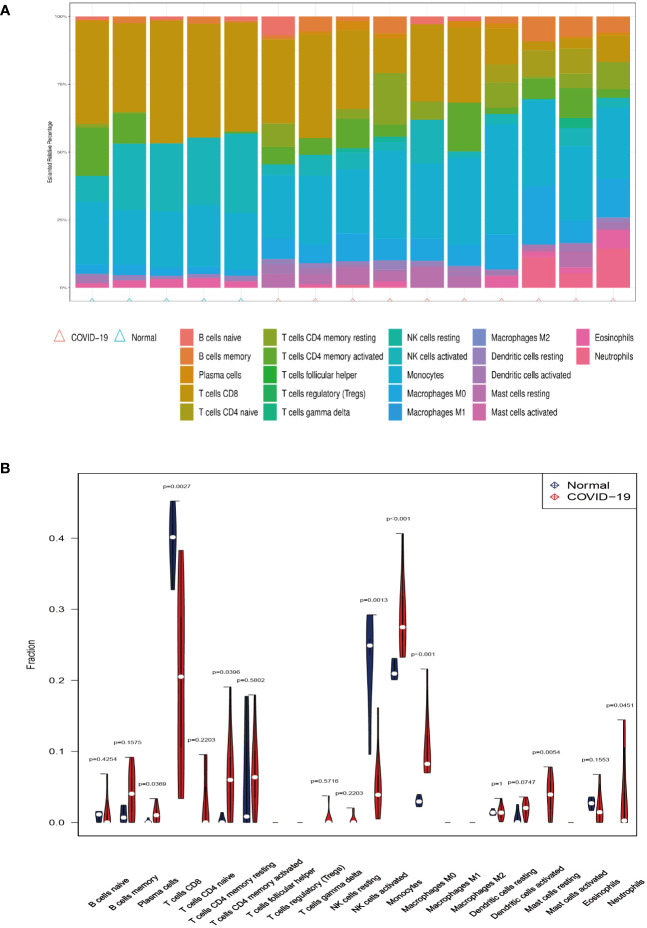
Immune infiltration analysis. **(A)** The ratio of 22 immune cells in COVID-19 and control. **(B)** The proportion and comparison of immune cells in COVID-19 and control.

**Figure 6:**
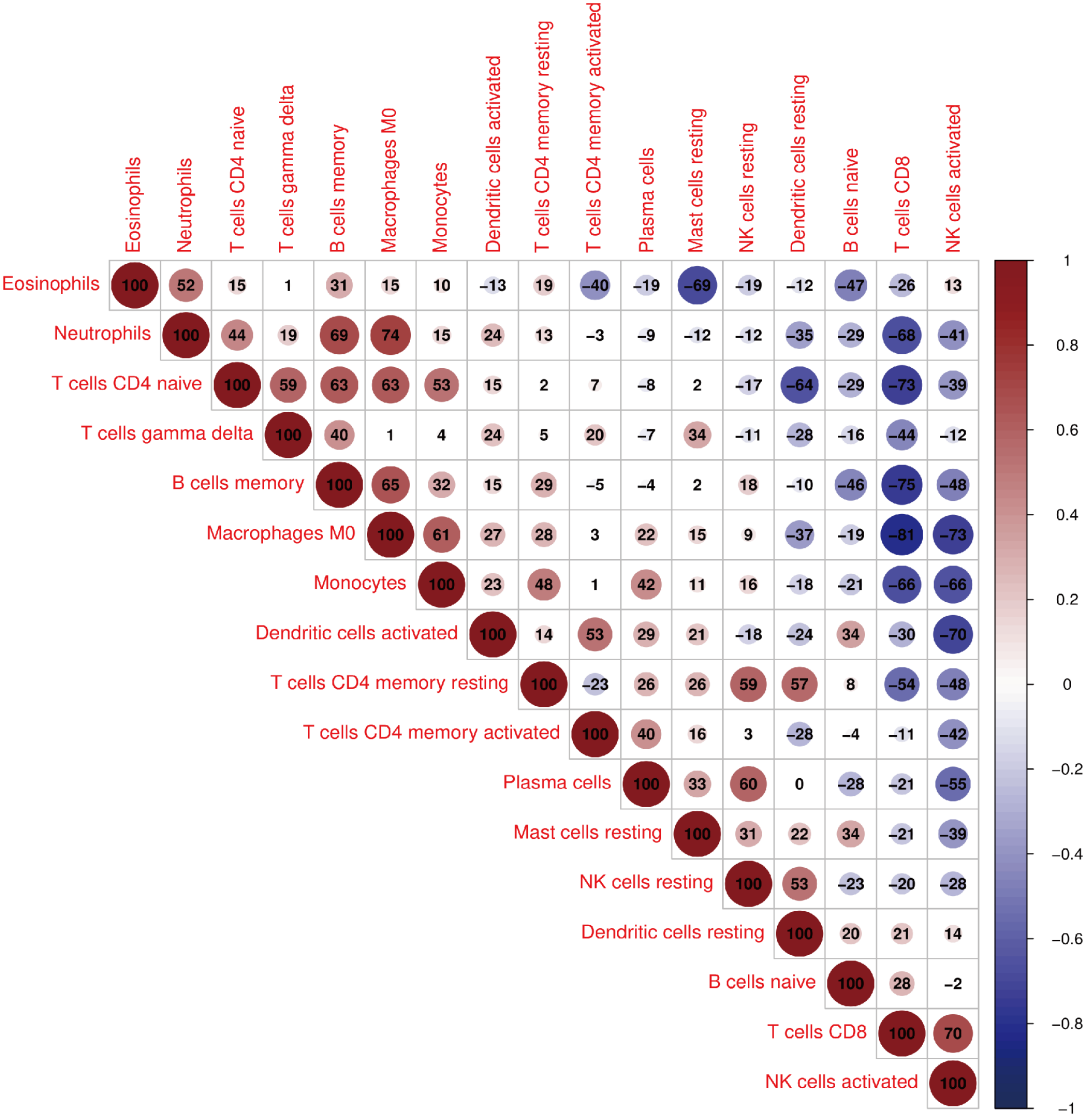
The correlation between the 22 immune cells in COVID-19.

Investigating immune cell infiltration patterns in COVID-19 patients was using the CIBERSORT algorithm. The proportions of 22 immune cells with COVID-19 are shown in **Figure 5A**. **Figure 5B** illustrates the distribution of 22 immune cells in COVID-19, and the infiltration of plasma cells, memory resting CD4 T cells, monocytes, M0 macrophages, resting mast cells and neutrophils in tissues from patients with COVID-19 is considerably higher than that in normal tissue (p < 0.05). Compared to the normal group, the proportion of activated CD8 T cells and natural killer (NK) cells in patients with COVID-19 is lower. Moreover, **Figure 6** depicts the relationships between the 22 immune cells. Neutrophils, naive CD4 T cells, memory B cells, M0 macrophages, and monocytes all showed negative correlations with CD8 T cells. NK cell activation was negatively correlated with M0 macrophage, monocyte, and dendritic cell activation. The relevance between neutrophils, memory B cells, and M0 macrophages was positive. Naïve CD4 T cells were positively related to gamma delta T cells, memory B cells, and M0 macrophages. Resting memory CD4 T cells and plasma cells showed the positive correlation with resting NK cells.

**Figure 7:**
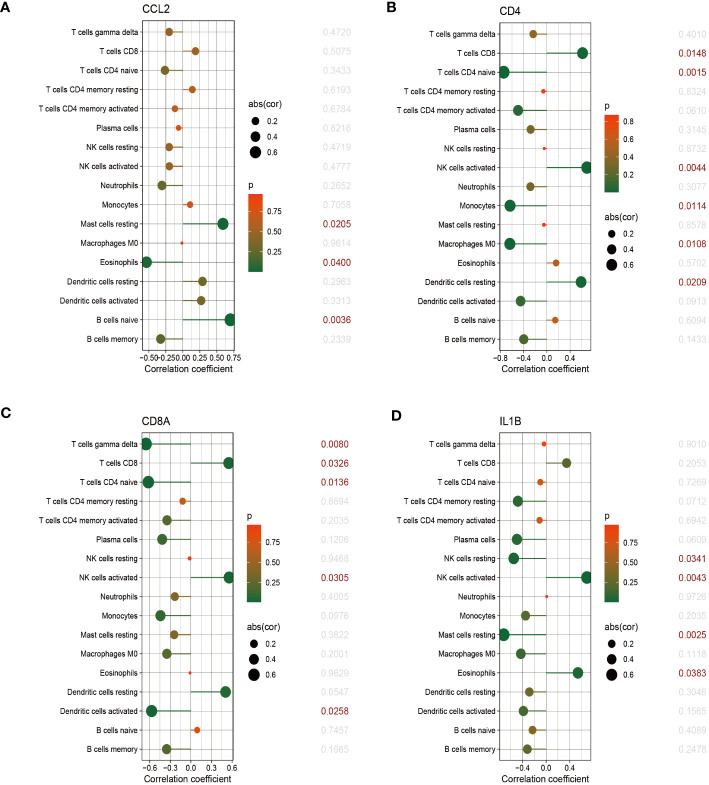
The correlation between hub genes and immune cells. The correlations of hub genes (CCL2, CD4, CD8A and IL-1β) with 22 immune cells were determined using p < 0.05 as the screening criterion.

**Figure 8:**
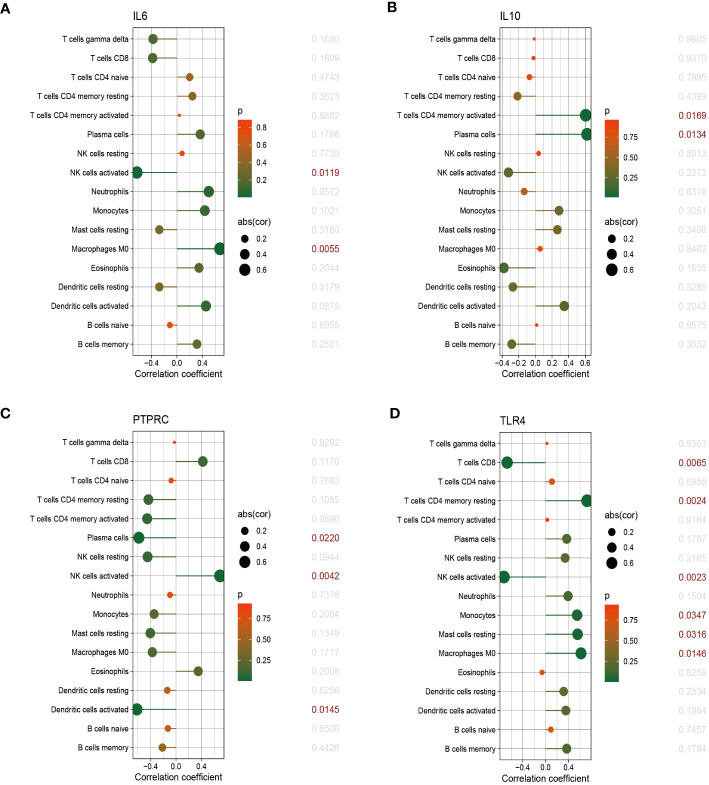
The correlation between hub genes and immune cells. The correlations of hub genes (IL-6, IL-10, PTPRC and TLR4) with 22 immune cells were determined using p < 0.05 as the screening criterion.

The relationship between immune cells and hub genes is depicted in **Figures 7**, **8**. *CCL2* exhibited a positive correlation with naive B cells and resting mast cells but a negative correlation with eosinophils. *CD4* was statistically positively correlated with CD8 T cells, activated NK cells and resting dendritic cells, but negatively connected with naïve CD4 T cells, monocytes and M0 macrophages. *CD8A* was statistically positively relevant to CD8 T cells, activated NK cells and resting dendritic cells, but negatively connected with gamma delta T cells, naïve CD4 T cells and activated dendritic cells. Activated NK cells and eosinophils statistically linked positively with *IL-1β*, but resting NK and resting mast cells statistically related negatively with *IL-1β*. Statistically, there was a positive correlation between *IL-6* and M0 macrophages but a negative association with activated NK cells. *IL-10* was statistically positively relevant to activated memory CD4 T cells and plasma cells. *PTPRC* was statistically positively associated with activated NK cells but, negatively correlated to plasma and activated dendritic cells. While CD8 T cells and active NK cells were inversely connected with *TLR4*, resting memory CD4 T cells, monocytes, resting mast cells, and M0 macrophages were positively related to *TLR4*.

### 3.5 Construction of gene regulatory networks

**Figure 9:**
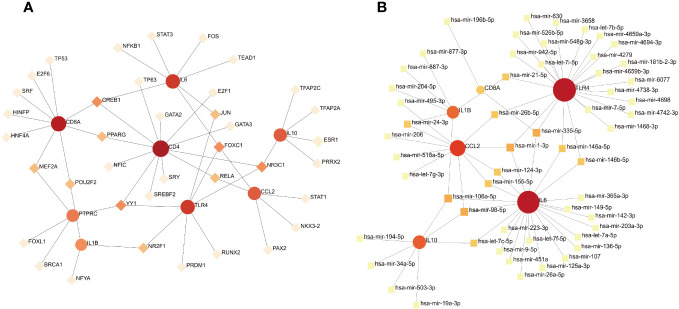
**(A)** TF–gene interaction network analysis. Dots represent hub genes; square dots represent transcription factors. Darker colors indicate stronger associations. **(B)** Gene–miRNA interaction network analysis. Dots represent hub genes; square dots represent miRNAs. Darker colors indicate stronger associations.

To identify the main variations at the transcriptional level and further study key protein regulatory molecules, we employed a network-based approach to decipher the regulatory TFs and miRNAs. 38 TFs were connected with the eight hub genes; these TFs included *MEF2A*, *POU2F2*, *CREB1*, *PPARG*, *YY1*, *NR2F1*, *JUN*, *FOXC1*, *NR3C1*, and *RELA* (**Figure 9A**). Six hub genes corresponded to 35 miRNAs; the miRNAs binding to multiple hub genes were hsa-mir-21-5p, hsa-mir-26b-5p, hsa-mir-24-3p, hsa-mir-335-5p, hsa-mir-1-3p, hsa-mir-146a-5p, hsa-mir-146b-5p, hsa-mir-124-3p, hsa-mir-106a-5p, hsa-mir-155-5p, hsa-mir-98-5p, and hsa-let-7c-5p (**Figure 9B**).

### 3.6 Gene–disease interaction network

**Figure 10:**
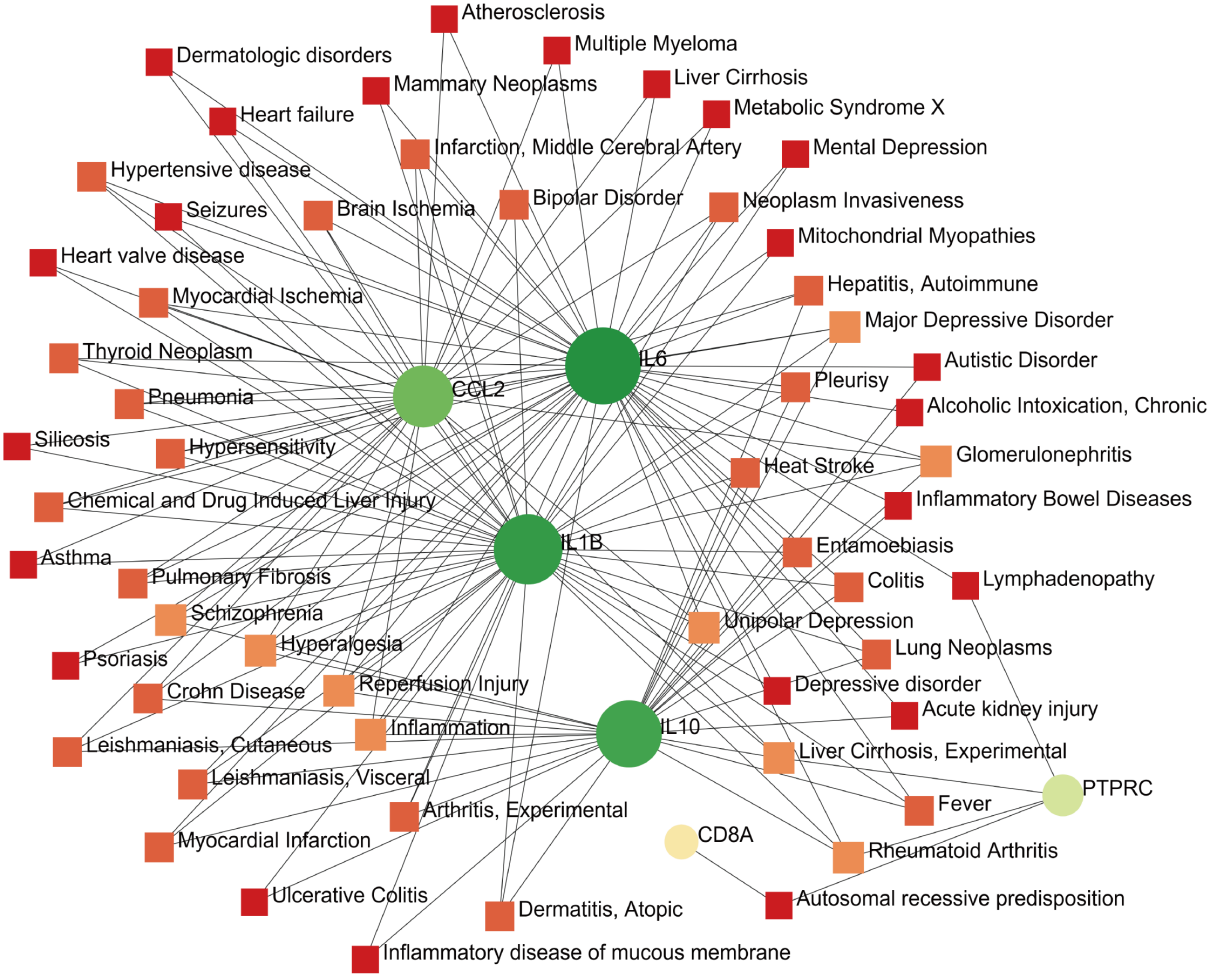
Gene–disease interaction network analysis. Dots represent hub genes; square dots represent diseases related to hub genes.

The development of technology and solutions for disease treatment begins with studying the links between diseases and genes; the interrelationships between different diseases usually require one or more similar genes (37). Based on DisGeNET, the results showed that the gene–disease network was linked to at least three hub genes. The following diseases had the strongest coordination with the hub genes studied: rheumatoid arthritis, glomerulonephritis, hyperalgesia, inflammation, liver cirrhosis, reperfusion injury, schizophrenia, and major depressive disorder (**Figure 10**). Notably, these diseases are mostly related to inflammation or immune responses, which have implications for the development of mechanisms and treatment methods for COVID-19 and pericarditis.

### 3.7 Protein–chemical and protein–drug Interaction networks

**Figure 11:**
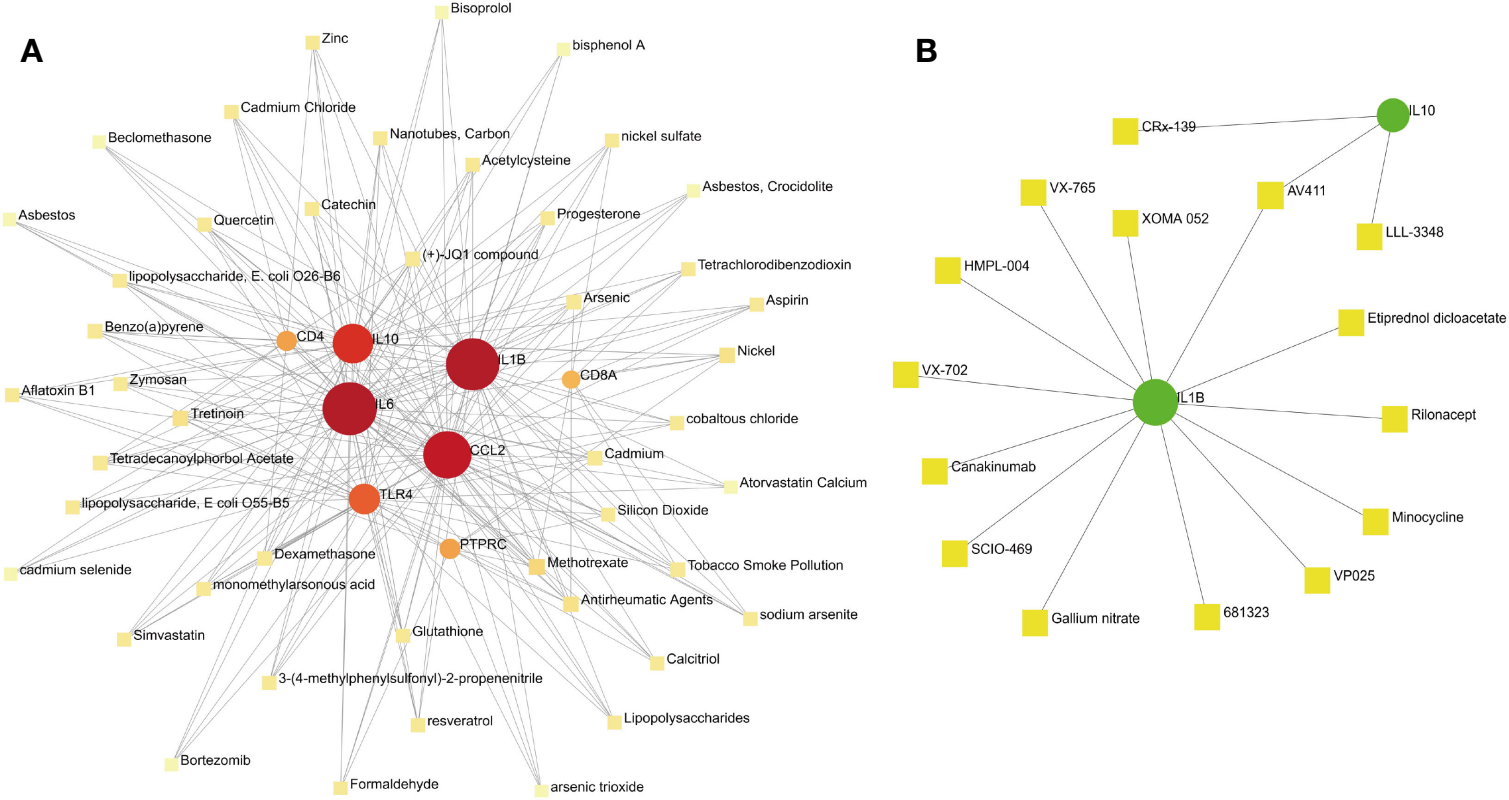
Protein–chemical and protein–drug interaction network analyses. **(A)** The interaction between hub genes and potential chemicals. **(B)** The interaction between hub genes and potential drugs. Dots represent hub genes; square dots represent chemicals or drugs.

Constructing protein–chemical and protein–drug interaction networks contributes to the exploration of the biological functions of proteins in cells and the research of potential drugs. Only the chemicals linked to at least four hub genes are displayed in **Figure 9**. The top eight chemicals were methotrexate, antirheumatic agents, nickel, tretinoin, arsenic, benzo(a)pyrene, cadmium, and dexamethasone, demonstrating their tight association with COVID-19 and pericarditis (**Figure 11A**). Protein–drug network indicates that drugs related to IL-1β and IL-10 may have broader scope for study, with AV411 having potential associations with two genes (**Figure 11B**).

## 4 Discussion

Increasing evidence has linked cardiovascular disease to increased morbidity and mortality from COVID-19, and the burden is evident even among patients who are not hospitalized (38, 39). COVID-19 is a real-time global pandemic, and this virus infection is also a pathogenic factor of pericarditis. COVID-19 has significantly increased the risk of pericarditis, and because multiple monitoring reports suggest that the COVID-19 vaccine may also increase the likelihood of people suffering from pericarditis, many studies on vaccines and pericarditis have been conducted in different countries and regions (40, 41). Currently, most reports on COVID-19 and pericarditis focus on the epidemiology and vaccines; however, research on the potential mechanisms of comorbidity remains lacking. Therefore, our research aimed to reveal the etiology and mechanism of COVID-19 and pericarditis from the perspective of molecular regulation, based on network data mining and bioinformatics analysis.

GO is a bioinformatics resource that provides gene product functions and uses ontology to represent biological knowledge and it can identify the biological process of shared genes in this study (42). The enrichment results in the BP and CC were associated with immune cell proliferation, including that of white blood cells, lymphocytes, and monocytes, suggesting their involvement in regulating cytokine production and inflammatory responses. Lymphocyte count and cytokine levels are closely correlated with disease severity, which is of great significance for the early diagnosis, treatment, and prognosis of COVID-19 (43). Gerd et al. examined the immune cell spectrum in the cerebrospinal fluid with COVID-19 and the results indicated an expansion of dedifferentiated monocytes and interferon signature of leukocytes (44). Anti-inflammatory treatment can reduce the thickness of pericardial late gadolinium enhancement, alleviate pericardial and systemic inflammation, and improve the physiological status and symptoms of pericarditis (45). Studies have focused on the prognostic value of inflammatory markers in active pericarditis, suggesting that high-sensitivity C-reactive protein may affect the intensity and duration of pericarditis (46). MF results mainly involve the binding of proteases and chemokines as well as the activity of signaling and immune receptors. Assessing the chemokine status after SARS-CoV-2 infection and detecting the “immune signature” is crucial for individual risk stratification (47). Studying the extensive cytokine releasing syndrome in COVID-19 may be helpful for targeting chemokines and growth factors as therapeutic drugs; furthermore, autoantibodies targeting chemokines may inhibit the potentially harmful immune response observed in patients with COVID-19 (48, 49). KEGG connects genomic and higher-order functional information from the perspective of genes and molecular networks and annotates up-to-date gene catalogs and functions (50). The top 20 pathways in KEGG involved immune-related pathways such as COVID-19, influenza, hepatitis, rheumatoid arthritis, and inflammatory bowel disease, and involved in immune pathways such as Th17 cell differentiation, IL-17, TNF, and Toll-like receptors. TNF-α blockers have made important progress in the treatment of idiopathic recurrent pericarditis (51). Christian et al. found that after virus clearance, clonally expanded Th17 cells remained in the lungs, which was associated with the potentially pathogenic cytokine expression profile of IL-17, and interacted with cytotoxic CD8 T cells and macrophages (52). As key regulatory factors of the innate immune system, Toll-like receptors recognize viral particles and induce the secretion of pro-inflammatory cytokines, which may also be potential targets for vaccine production (53). In this study, we constructed the gene network to obtain the common differential genes of COVID-19 and pericarditis, so as to obtain the hub gene and explore the potential mechanism correlation between them. GO term functional enrichment and KEGG pathway enrichment were applied to identify functional changes caused by differential genes and their effects in the pathway. However, due to the need for a clear threshold for GO/KEGG enrichment of the common genes, it is possible to miss genes with significant biological significance. GSEA, which may not require the clear threshold and is based on overall trend analysis, can be implemented in the future. Meanwhile, Weighted correlation network analysis (WGCNA) can serve as a supplement to gene network studies and can analyze gene modules that coordinate expression.

Immune cell infiltration showed that patients with COVID-19 had significantly higher levels of plasma cells, resting memory CD4 rest cells, monocytes, M0 macrophages, resting mast cells, and neutrophils than the healthy population. Persistent antibody protection, produced by memory B cells and long-lived plasma cells, is the pillar of the “arms race” between vaccines immunity and the constantly mutating SARS-CoV-2 virus infection, and long-lived plasma cells in bone marrow tissue are the source of these persistent “memory” antibodies during acute infection (54). Some studies have evaluated whether pre-existing cross-reactive memory T cells affect vaccine immunity, and the results displayed that subjects with memory CD4 T cells have stronger antibody responses to vaccines (55). Notably, long-lasting memory T cells responded to SARS-CoV-2 and exhibited substantial cross-reactivity with the N protein of SARS-CoV-2 in patients recovering from SARS-CoV-2 in 2003 (56). Excessive infiltration of macrophages and monocytes into organs is a critical driver of severe COVID-19, and the activation of pulmonary macrophages from infiltrating monocytes results in the recruitment of cytotoxic effector cells and the release of pro-inflammatory cytokines (57). Macrophages activate inflammasomes, which oppose host infection and promote tissue repair by releasing interleukin and inducing pyroptosis; however, it should be noted that macrophage activation syndrome induced by macrophage dysfunction may cause damage to the host (58, 59). The high density of mast cells is related to the activation and release of proteases, which are affected by soluble factors released by T cells with the help of stem cell factors. The proliferation and activation of mast cells are manifestations of inflammatory cell changes in severe and lethal SARS-CoV-2 infection; thus, regulating mast cells and their pro-inflammatory mediators may be a potentially effective treatment for COVID-19 (60, 61). The characteristics of neutrophils in severe COVID-19 include the formation and degradation of neutrophil extracellular traps, expansion and infiltration of neutrophils into the lungs, and activation and immune suppression of neutrophil subsets in the circulatory system (62). The abnormal response of neutrophils after infection with SARS-CoV-2 may be related to uncontrolled viral replication and exacerbated inflammation. Assessing the number, function, and status of neutrophils are crucial for distinguishing the disease severity and identifying the clinical deterioration risk (63). Many studies have been conducted on the progression of immune cells in pericarditis. Neutrophils and macrophages produce a large number of cytokines through the activation of inflammasomes, which contribute to the immune pathogenesis of recurrent pericarditis (64). Interference with neutrophil chemotaxis and adhesion, reduction of recruitment to damaged tissues, and superoxide production are effective strategies and mechanisms for the anti-inflammatory treatment of pericarditis (65). The pericardial interstitial cells of patients with pericarditis exhibit senescent features that induce structural remodeling of the pericardium, such as increased collagen matrix secretion and calcium deposition, promotion of chemotaxis of monocytes/lymphocytes, and recruitment of inflammatory factors (66). Moreover, many clinical samples have been collected from patients with pericarditis. Pleural biopsy revealed pleural pericarditis accompanied by lymphoplasmacytic inflammation, such as IgG4-positive plasma cells, and detection of pericardial fluid indicated that the histamine receptor depended on mast cells to infiltrate the pericardial tissue and was involved in the inflammatory reaction (67, 68). Notably, this study analyzed the difference of immune infiltration in COVID-19 and identified the types of immune cells associated with hub gene. With the accumulation of research data related to pericarditis, immune infiltration analysis of pericarditis can be carried out in this study.

Based on the PPI network and topological analysis, *IL-1β*, *CD8A*, *IL-10*, *CD4*, *IL-6*, *TLR4*, *CCL2*, and *PTPRC* were identified as hub genes. Since the COVID-19 pandemic, many trials have found that the COVID-19 group had higher levels of IL-1, IL-6, and IL-10 than the control group. Logistic regression and ROC analyses have revealed that these cytokines have a predictive effect on disease severity (69, 70). A genome-wide association study showed that patients with critical COVID-19 had significantly greater blood IL-6 expression levels than patients without symptoms and that an allele change at the rs2069837 site can reduce IL-6 levels to prevent critical conditions (71). Anti-interleukin (IL)-1 drugs have been developed and used to treat autoimmune and rheumatic immune diseases. As one of the family members with the strongest pro-inflammatory effects, IL-1β is considered the therapeutic target for recurrent idiopathic pericarditis (72). Research has been conducted on the etiology, immune mechanisms, and treatment of tuberculous pericarditis, and showed that IL-10 levels are elevated in the pericardium and blood (73). Myocardial fibrosis is considered a non-negligible feature of constrictive pericarditis. IL-6 mediates abnormal Ca^2+^ handling and induces atrial fibrosis in sterile pericarditis rats (74). Patients with severe COVID-19 exhibit less pronounced increases in TLR4 expression on CD14 monocytes than those with mild COVID-19, which is related to activation of TLR4/NF-κB axis after lipopolysaccharide stimulation (75). TLR4 signaling pathway also regulates myocardial fibrosis by inhibiting its target genes (76). The detection of specific chemokines in the plasma at the mRNA and protein levels suggests that higher concentrations of CCL2 are associated with the severity of COVID-19, which has potential as a prognostic factor (77). The immunopathological changes in the spleen of patients with COVID-19 are also worthy of attention as they involve the functions of plasma cells and monocytes/macrophages and a decrease in CD8A abundance (78). Through the analysis of transcriptome data, PTPRC was shown to be an important inflammatory and immunomodulatory signature in COVID-19, and that it has high binding efficiency with related drugs in clinical transformation research (79). There have been some studies on CCL2, CD8A, and PTPRC in cardiovascular diseases; however, their roles in pericarditis require further research. Overall, these hub genes may be potential immune regulatory pivots in COVID-19 and pericarditis. In addition, there is a very interesting issue worth discussing. The expression of pro-inflammatory cytokines such as IL-1β and IL-6 may change over time, and their dynamics may be potential predictors of disease (80). Currently, database-based research considers gene expression at different time points as a whole, and more in-depth studies in the future will focus on dynamic changes in gene expression to achieve precise intervention for diseases.

We constructed TF-gene and gene-miRNA interaction networks to better understand the molecular regulation between COVID-19 and pericarditis. *CREB1*, *YY1*, *FOXC1*, and *NR3C1* were the TFs having the strongest correlation to the hub genes. We analyzed the transcriptome RNA-seq data related to COVID-19 and used bioinformatics to decode the molecular tags and pathways of the host cell response to SARS CoV-2. The genes *YY1* and *CREB1* may co-regulate autophagy to affect severe conditions. *FOXC1* and *YY1* may have good binding affinities to candidate drugs. The NR3C1-CXCL8-neutrophil axis may determine the severity of COVID-19 (81–83). The miRNAs that strongly interacted with the hub genes were hsa-mir-335-5p, hsa-mir-1-3p, hsa-mir-106a-5p, and hsa-mir-98-5p. In different studies, peripheral blood mononuclear cells, serum samples, and bronchial aspirates from patents with COVID-19 and healthy individuals were collected. Sequencing analysis has shown that miR-1-3p is involved in the regulation of autophagy and has high specificity and sensitivity for predicting mortality (84, 85). MiR-335-5p is regulated by angiotensin-converting enzyme and histone deacetylase and is involved in drug development to interfere with host-virus interactions (86). *TMPRSS2* is a potential therapeutic target for COVID-19, and miR-98-5p is a regulatory factor of *TMPRSS2* that originates from two types of endothelial cells in the lungs and umbilical vein (87). Due to the lack of transcriptome sequencing and network information analysis of pericarditis, the regulatory roles of these TFs and miRNAs in pericarditis need to be further elucidated.

According to the gene-disease interaction network, COVID-19 combined with pericarditis can damage the heart, liver, kidney, and other organs; trigger inflammation and rheumatism; and cause neurological and psychiatric diseases. Several cases have reported that simultaneous onset of glomerulonephritis and pericarditis in patients with rheumatic immunity and viral infection (88, 89), and many studies have focused on COVID-19 vaccine that may increase the risk of glomerulonephritis similar to pericarditis (90, 91). The humoral immune response of patients with liver cirrhosis after COVID-19 vaccination is being explored, and case suggest that constrictive pericarditis may appear as a comorbidity in patients with liver cirrhosis (92, 93). The increased incidence of rheumatoid arthritis in patients with COVID-19 may be due to the impaired function of the autoimmune system and the iatrogenic effect of immunosuppressants, and genome-wide cross-trait analysis shows that higher genetic susceptibility to rheumatoid arthritis also increases the risk of COVID-19 (94, 95). A similar mechanism reveals the possibility of antirheumatic drugs as a potential treatment for COVID-19. The effects of drugs on pericardial contractions secondary to rheumatoid arthritis have also been studied in patients with pericarditis (96, 97). Globally, major depressive disorder and anxiety disorder cases have increased by 27.6% and 25.6%, respectively, as a result of the COVID-19 pandemic, resulting in 49.4 million and 44.5 million DALYs (disability-adjusted life years) (98). Immune dysfunction caused by infection can aggravate mental sequelae, and studies on the influence of COVID-19 on mental health have found that the levels of inflammatory markers are directly proportional to depression severity of depression (99). Tryptophan metabolism may be correlated with the potential susceptibility to depression, and tryptophan supplementation may improve depressive symptoms in patients with COVID-19 treated with drugs that can affect tryptophan metabolism (100). Numerous investigations have shown that COVID-19 increases the risk of schizophrenia, and willingness to be vaccinated is related to the severity of psychiatric symptoms (101, 102). A case report of clozapine-related pericarditis in a patient with refractory schizophrenia during the drug titration phase suggested that great attention should be paid to the side effects of antipsychotics and antidepressants in patients with COVID-19 and pericarditis (103).

Protein–chemical interaction networks indicated that methotrexate, antirheumatic agents, nickel, tretinoin, arsenic, benzo(a)pyrene, cadmium, and dexamethasone have a high correlation with hub genes. As a specific immunosuppressive drug, methotrexate impairs immunogenicity and raises the risk of infection and poor prognosis (104). Interruption of methotrexate for two weeks enhances antibody responses in patients with immune-mediated inflammatory diseases after vaccination (105). IL-6 and IL-1β are pivotal targets of antirheumatic agents, and there is evidence that blocking the IL-6 receptor can reduce lung involvement and acute cardiovascular complications in patients with COVID-19 by inhibiting the systemic inflammatory response (106). Several clinical trials have been conducted to evaluate the long-term prognosis of COVID-19 with different doses of dexamethasone and whether the clinical benefits are related to different respiratory support modes (107, 108). Methotrexate has previously been used for the treatment of purulent pericarditis in rheumatoid arthritis; however, methotrexate-induced pericarditis and pericardial effusion should be considered (109, 110). In addition, clinical guidelines indicate that non-steroidal anti-inflammatory drugs (NSAIDs) such as aspirin are recommended as effective drugs for the first-line treatment of pericarditis (111). Notably, when the screening scope of the interaction network is expanded, NSAIDs such as aspirin and ibuprofen can also be searched. Protein–drug interaction networks revealed that some drugs, such as AV411, minocycline, rilonacept, canakinumab, XOMA 052, and VX-765, exert therapeutic effects by targeting hub genes. AV411 reduces opioid withdrawal by inhibiting glial pro-inflammatory responses, whereas minocycline prevents potentially fatal arrhythmias by inhibiting pro-inflammatory cytokines and poly (ADP-ribose) polymerase-1 associated with SARS-CoV-2 replication (112, 113). Rilonacept is a trap for IL-1β and has been shown in clinical trials to inhibit recurrent pericarditis episodes and prevent the recurrence of pericarditis (114). Canakinumab, a human monoclonal antibody targeting IL-1β, is associated with the reduction of serum C-reactive protein level and the improvement of overall mortality in COVID-19; case reports showed that canakinumab can reduce the risk of recurrence of systemic disease-related pericarditis (115, 116). As the neutralizing antibody to IL-1β, XOMA 052 has a rapid onset and sustained control of intraocular inflammation, and VX-765 ameliorates myocardial reperfusion injury by inhibiting caspase-1 activity and reducing lactate dehydrogenase release (117, 118). In summary, some chemicals and drugs predicted based on hub genes have been proven in clinical trials and experimental studies of COVID-19 and pericarditis, whereas others deserve further exploration.

This study had several limitations. There are currently no suitable microarray or RNA sequencing data for pericarditis, resulting in a lack of available datasets that may prevent the acquisition and identification of sufficient DEGs. In addition, our study was purely based on bioinformatics analysis and requires subsequent *in vivo* and *in vitro* to confirm the validity of the results, as well as to fully evaluate the biological function of the hub gene and the clinical value of the drug.

## 5 Conclusion

In recent years, the topic of increased risk of pericarditis caused by COVID-19 has triggered a large number of studies and heated discussions, but there is still a lack of exploration and research on the mechanism of COVID-19 and pericarditis. The immunological mechanisms and common genes linked to COVID-19 and pericarditis were identified in this investigation. The eight hub genes (*IL-1β*, *CD8A*, *IL-10*, *CD4*, *IL-6*, *TLR4*, *CCL2*, and *PTPRC*) are relatively mature and have been extensively studied in immune regulation, and some also have the potential to affect immune functions. Thus, COVID-19 and pericarditis exhibit complex interactions. The enrichment analysis and various interaction networks constructed and analyzed in this study revealed the molecular mechanisms of COVID-19 and pericarditis from multiple perspectives. Based on the analysis, some potential compounds and drugs were predicted. However, further research on their functions and mechanisms is required to provide new ideas for identify potential biomarkers and explore appropriate treatment methods.

## Data availability statement

The original contributions presented in the study are included in the article/**Supplementary Materials**, further inquiries can be directed to the corresponding authors.

## Author contributions

DL: Formal Analysis, Investigation, Resources, Validation, Visualization, Writing – original draft, Writing – review & editing. RC: Visualization, Writing – review & editing. CH: Writing – review & editing. GZ: Writing – review & editing. ZL: Writing – review & editing. XX: Writing – review & editing. BW: Writing – review & editing. BL: Writing – review & editing, Writing – original draft. XC: Writing – review & editing, Writing – original draft.

## Supplementary material

Supplementary Figure 1 Schematic overview for gene set enrichment analysis. GSEA is a computational method that determines whether an *a priori* defined set of genes shows statistically significant, concordant differences between two biological states. An expression data set sorted by phenotype, heat map and the gene tags, and plot of the running sum for dataset, including the location of the maximum enrichment score (ES) and the leading-edge subset (https://www.gsea-msigdb.org/gsea/) (16).
