# Supplementary figures and images for: Comprehensive bioinformatics analysis and systems biology approaches to identify the interplay between COVID-19 and pericarditis
Source: Front Immunol. 2024 Feb 22;15:1264856. doi: 10.3389/fimmu.2024.1264856 (PMC10918693; doi:10.3389/fimmu.2024.1264856)

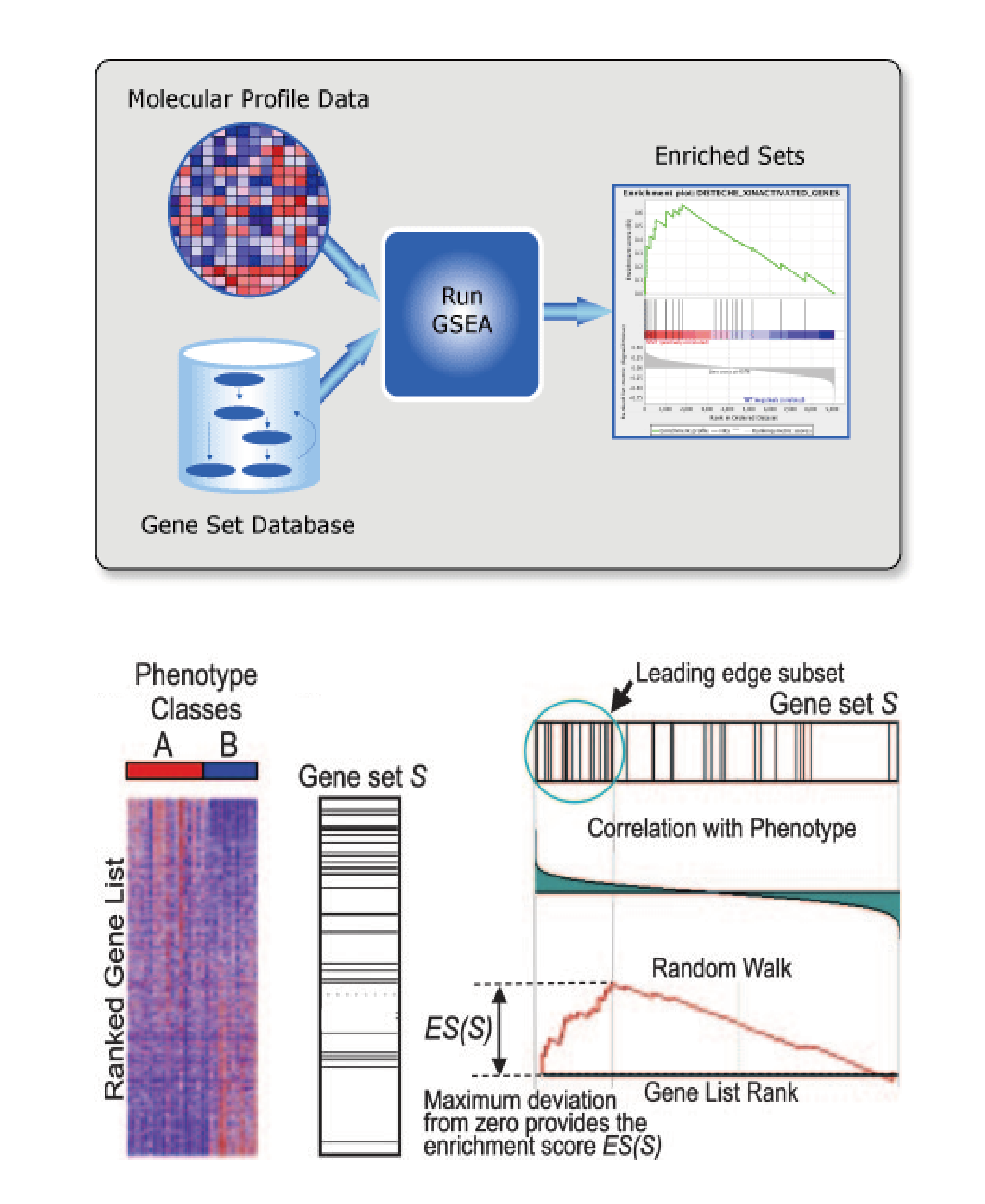

Supplement: Supplementary Figure 1 — Schematic overview for gene set enrichment analysis. GSEA is a computational method that determines whether an a priori defined set of genes shows statistically significant, concordant differences between two biological states. An expression data set sorted by phenotype, heat map and the gene tags, and plot of the running sum for dataset, including the location of the maximum enrichment score (ES) and the leading-edge subset (https://www.gsea-msigdb.org/gsea/) (16). [file Image_1.tif]
