# Supplementary material for: Comprehensive bioinformatics analysis and systems biology approaches to identify the interplay between COVID-19 and pericarditis
Source: Front Immunol. 2024 Feb 22;15:1264856. doi: 10.3389/fimmu.2024.1264856 (PMC10918693; doi:10.3389/fimmu.2024.1264856)
Supplement: Supplementary file 2 [file Table_1.doc]

TABLE S1 COVID-19-related genes after filtering.

| GeneCards (n=500) | DisGeNET (n=500) | CTD (n=500) | GEO (n=494) |
| --- | --- | --- | --- |
| ACE2  TLR7  TMPRSS2  IL6  IFNAR2  TNF  ACE  CRP  FURIN  CXCL10  CXCL8  IL1B  CCL2  IL10  NLRP3  F3  NRP1  CGAS  TNNI3  IL2RA  DPP4  F2  IL2  IFNB1  IL4  HLA-A  STING1  CTSL  CCR6  CCL3  AGT  TYK2  REN  HLA-C  IFNA2  TLR4  VWF  AGTR1  IFNA1  IRF3  SLC6A19  VDR  IL17A  ALB  CD8A  NPPB  AGTR2  STAT2  EGFR  LOC117134593  IL22  TMPRSS4  IFNG  PLG  TBK1  NRP2  NFKB1  GPT  SERPING1  IL7  KNG1  CSF2  LOC117134604  LOC117134608  LOC117135106  IL18  LOC112679198  OAS1  BMAL1  TNFRSF1A  NOS3  BSG  TNFRSF1B  LOC117134605  LOC117134606  CD4  INS  IFNAR1  NFKBIA  FCGR2A  BDKRB1  SERPINA3  RELA  MIR146A  TLR3  FCGR3A  APOE  CSF3  STAT1  MUC1  SERPINE1  CCR5  IFITM3  ADAMTS13  FCGR3B  MBL2  ABO  CALCA  S100A9  HSPA5  TAMM41  LZTFL1  SH2D3A  HLA-B  IL10RB  AR  ADAM17  HMGB1  SLC6A20  S100A8  SFTPD  HMOX1  JAK1  CHAT  PMS2  INPP5E  HIF1A  MIR21  STAT3  HLA-DRB1  ADM  CD274  IFIH1  MIR155  LINC00562  TREM1  SERPINA1  CXCR6  G6PD  PDCD1  SCARB1  SIGLEC5  LOC119086083  LOC126806670  FYCO1  ANGPT2  PTX3  NEAT1  RAB7A  TGFB1  MUC5B  CST3  CD209  CD14  IL6R  SERPINC1  ISG15  DPP9  TLR2  THBS3  CD163  LGALS3  ELF5  APOL1  AAK1  VPS39  LOC117134607  LOC117134611  LOC117135104  LOC117135105  SELP  CCR9  ELANE  LOC110121154  LOC127276353  LOC127400449  LOC127819970  LOC127820730  LOC127825881  LOC127826279  LOC127884359  LOC127885445  LOC127888870  LOC127890069  LOC127891453  LOC127891703  LOC127891910  LOC127892477  LOC127893260  LOC127894021  LOC127896910  P2RX7  IL13  ENPEP  IRF7  AGER  ACAT1  HAVCR2  VEGFA  BDNF  MALAT1  PRF1  GDF15  FAS  IL33  ESM1  CCL5  ZC3HAV1  SIGLEC1  MIR126  CFTR  RAVER1  CD28  CLEC4M  MUC5AC  FGA  IGF1  AXL  IL1A  TNNT2  PLAUR  OAS2  OAS3  CHI3L1  AVP  ATP11A  CCL4  CCL7  NFE2L2  TLR9  CTLA4  THBD  FOXP4  TAC4  F13A1  FGB  LDHA  PLSCR1  MPO  LOC118966792  TAS2R38  CCR2  XCR1  CCR1  CCL11  ENTPD1  TLR8  HLA-DOB  NEU1  FUT2  BCL11A  EFNA4  RGMA  ACSL6  KANSL1  TRIM46  IFNA10  ZGLP1  SLC22A31  LINC00649  LINC01276  FLT1  SFTPB  SFTPC  LOC117600004  VPS41  GZMA  HLA-G  UGT2A1  UGT2A2  CXCL1  LPA  ANXA2  IKBKB  IFNL1  VAMP8  BTK  TTR  ALOX5  NFKBIZ  MTHFR  F11  SIGMAR1  EDN1  C5AR1  IRF9  IFNL4  CTSB  IDO1  KIR3DL1  ERVW-1  CNR2  LINC02967  VPS11  MAS1  ANGPTL4  ADAM9  FABP2  IFITM1  IFNL2  SOCS1  SNAP29  STX17  LCN2  SDC1  MYD88  TAS2R1  GOLGA3  AKAP8L  BECN1  C5  IDO2  IFI44  MX1  SCGB1A1  RIGI  GSTT1  EXOSC2  RIOX2  MOV10  IL15  CXCL2  RAB8A  FNDC5  IL2RB  SAA1  MIR320B1  STAT5B  FGG  STAT5A  FOXP3  CD80  CD86  F13B  LRCOL1  UBAP2L  RHOA  SLC6A4  PIRAT1  PROCR  GC  MAVS  ERG  MIR142  RPL36  CD99  AP3B1  RNH1  CXCL9  NR3C1  ATF4  MMP1  GPX1  DNMT1  SDCBP  AKT1  AHR  WWP2  UBC  EIF2B1  THRIL  IFNGR1  IFNA6  TES  VKORC1  BRD4  CCL17  SLC6A15  EIF3G  NEDD1  IKBKG  HAVCR1  WWP1  IFNL3  LMNB1  MIR29A  IL1F10  MTX1  SLC25A1  GAS6  RBM15  PAPPA  SELL  MT-ATP6  FBXL12  KLRC1  ICAM1  CCR3  MIR150  STX7  STX6  VPS16  VPS18  PTBP1  RPL13A  BRD2  IKBKE  G3BP1  G3BP2  SMN1  ORAI1  EPO  CNBP  AKAP8  NCAM1  CXCL16  LOC106699567  TH2-LCR  LOC111832671  LOC112590816  LOC112590817  LOC116158494  LOC116158495  LOC116183086  LOC116183087  LOC117152610  LOC117152611  LOC117204000  LOC117204001  LOC117693187  LOC119230225  LOC119266102  ATP2B1  PIGS  RPS18  KLF2  NEDD4  PAFAH1B1  C9orf72  JAK2  ICOSLG  TEK  STAT6  SIRT3  EPHA7  PALM  SURF6  TF  PIK3C3  ROCK1  FASN  TIMM29  ANXA1  MIR200C  MIRLET7B  UNC93B1  ILF3  F10  HSPA1A  SOD2  DEFA1  PLA2G7  ITGAV  VPS33A  PALS1  TOMM70  PLA2G2A  DCLK1  PGF  IL6ST  TRPV1  TRPA1  GSTM1  IFNAR2-IL10RB  CHD3  HSD17B4  DDX20  RPS15  LRRFIP2  TPM4  MLEC  MON2  S100A4  S100A10  SCUBE1  CHRFAM7A  TUG1  MIR139  MIR30A  SNHG16  SNHG6  ITK  CAT  SIRT1  SMURF1  INHBA  SMURF2  INHBB  PSME3  RAE1  CNR1  ADIPOQ  S100A12  PTPRJ  HMGCR  ADAR  PDHX  CYB5A  AGPAT1  DPM3  FAT1  PDS5A  PEG10  SACM1L  PLXNB2  SEC16A  TBC1D5  MYDGF  TRAFD1  PON1 | ACE2  CRP  S  IL6  ORF1ab  ACE  REN  TNF  TMPRSS2  CD4  CD8A  AGT  ALB  IL1B  GPT  F2  IL10  LOC102724971  LOC102723407  INS  IFNG  N  CXCL8  AMH  IL1A  IFNA1  FURIN  CSF2  DPP4  TNNI3  E  F3  IL2  GOT1  CRX  IFNB1  NFKB1  AGTR1  RPGR  PDB1  IL6R  PLAT  CALCA  CCL2  IL17A  SERPINA13P  HLA-C  LSAMP  LAMP3  ABO  HBA1  CENPJ  IL2RA  NELFCD  MB  CXCL10  VWF  CTSL  NLRP3  ORF8  CTRL  CTSB  IL4  SPECC1  MAS1  ZFYVE9  MS4A1  PDCD1  RTN1  RTN4  BSG  MTOR  NCKIPSD  AHI1  SOAT1  VEGFA  PLG  KRT20  F8  JAK1  SERPINA5  CD19  IFNA2  PSMD1  STAT3  TTR  KNG1  NPPB  EMSLR  AGTR2  CSF3  IL7  IL18  PROS1  LINC01672  ESR1  NFE2L2  PSS  ORF3a  C5  EEF1A2  ERBB2  G6PD  GGT1  NCAM1  ADAM17  TLR4  GGTLC5P  GGTLC3  GGT2  GGTLC4P  FCGR3A  FCGR3B  HSPA5  IL1RN  INSRR  JAK2  CCL3  CCL5  TLR3  KLK4  SH2D3C  BTK  VPS51  ABCB1  WDTC1  PGR-AS1  C3  CD14  F10  HIF1A  HLA-A  SERPINF2  SLC5A2  ADAMTS2  TLR7  STS  CYP3A4  FLT4  HMGB1  LTF  EPCAM  COX2  PRF1  MAPK1  PKD2L1  SLC33A1  ADAMTS13  IGKV2D-29  PYCARD  ASZ1  M  ORF6  ORF7a  AR  CASP1  CDSN  CEL  HLA-B  IGHE  IL13  LCT  MME  MUC1  OCA2  PRKAA2  STAT1  B3GALNT1  RHOD  SCPEP1  FUZ  SCAI  ORF10  MS2  AKT1  C5AR1  CASP3  CD48  CD68  EGFR  DMTN  EPHA3  EPO  ICAM1  JUN  MBL2  MEFV  MPO  PRKAA1  PRKAB1  NECTIN1  SELP  TFPI  APOL1  RNMT  HGS  ISG15  CABIN1  CD2AP  CD274  IL23A  CDCA7L  OTOR  IFIH1  SLTM  OMA1  SLC6A19  SERPINA3  ACR  PARP1  AGER  ANGPT1  ANPEP  AQP4  CD38  CDKN3  CCR5  CRYGC  CYP2D6  ATN1  ELANE  FBL  GLP1R  HLA-DRB1  HMOX1  MET  NR3C2  MMP9  MUC5AC  MX1  NEU1  OXT  SERPINE1  PIK3CA  PIK3CB  PIK3CD  PIK3CG  PPARG  PROC  MOK  SAA1  NRP1  NAPSA  SH2D3A  IFITM3  PITRM1  COPE  DDX58  PLA2G15  NAAA  BBS9  FAM3B  ACSS2  ACCS  CRYGEP  A1BG  KLK3  FAS  CD40LG  CECR  CPOX  CSF1  SLC25A10  DAPK3  DHODH  DIO3  EPHA2  FUT3  GCG  NR3C1  HBB  HBG2  CFI  IGF1  ISG20  ITGAM  IVD  KLRC1  LEP  LRP2  MMP8  NOS2  NPC1  MAPK8  PTGS2  PTPRC  TF  UMOD  AD5  SKAP2  PPIG  RAPGEF5  CTPP  CLEC4M  CPQ  SLC27A5  SPACA9  MCF2L  SIRT1  SETD2  FEV  PARP9  ZGPAT  GGTLC1  COPD  ORF7b  LOC102724197  ABL2  AHR  AMBP  ANG  APEX1  APP  ARSL  ASIP  AVP  OPN1SW  BCS1L  BRCA1  CAD  CALR  CFTR  CHAT  COL11A2  CRYGD  DNAH8  EPHB2  FLOT2  FLT1  FN1  GAST  GAD2  OPN1MW  GFAP  GH1  CXCR3  CXCL1  GZMB  HOXB5  IL5  ITGAX  KRT12  LPO  CXCL9  MRC1  NT5E  OAS1  PAEP  PCOS1  PF4  SERPINA1  PKM  PON1  PPARA  PRH1  PRH2  PLAAT4  BRD2  RPS6KA1  RTN2  S100A8  CCL7  CCL20  SECTM1  SLC3A2  SMARCB1  SRY  TBXT  TLR2  TWIST1  USF2  VDR  CXCR4  BCAR3  NR1I2  CD163  ABCB6  SPAG5  CXCR6  FASTK  PRAME  ARIH1  IL37  IGKV7-3  CD209  IL22  TLR9  SAGE1  CCDC88A  NKRF  TMPRSS4  CD177  HAMP  PAGR1  IL1F10  HAVCR2  UBXN11  DNER  CARD16  MRGPRD  SYNPR  PIKFYVE  PCSK9  SLC26A5  SMIM10L2A  NPS  SPANXB1  ACOD1  ERICD  ADM  ADRA1A  ADRA2B  AKR1B1  ANXA13  APC  APOA1  APOE  ATR  BCHE  BCR  BRAF  BRS3  TSPO  CA2  CAMP  CAV1  CD74  CEACAM5  CTSC  CPE  CST3  CTLA4  DDIT3  DNASE1  EDNRA  EGF  ENPEP  ERG  F7  FOLH1  FUT1  GALNS  GC  GOLGB1  GPR42  HK1  HP  HPN  IFIT1  IGF1R  IL1R1  IRF1  IRF7  ITGA2B  ITGB2  ITGB3  KLKB1  KRT10  KRT18  LY6E  MAX  MNT  MOG  ABCC1  NEFL  OAS2  OPRD1  PDE4A  PGF  SLC25A3  PIP  POMC  PPIA  MAPK3  RAF1  REL  RPS6KB1  S100A12  CCL4  CCL8  CCL11  SELE  SELENOP  SLC6A1  SOD1  TRIM21  SSTR4  TAPBP  TRBV20OR9-2  TGFB1  THAS  THBD  TLE1  TLE2  TLE3  TLE4  TLR5  SGCE  LPAR2  TMPRSS11D | TP53  CCL2  IL6  NFKBIA  TNF  RELA  CXCL8  IL10  NFKB1  CASP3  NOS2  CASP8  BAX  ABCB1  PCNA  BCL2  AKT1  CDKN1A  BBC3  IL1B  ICAM1  SERPINE1  PARP1  IL1A  SQSTM1  SOD2  FOS  IFNG  COL1A1  PPARG  HSPA5  TGFB1  CYP3A4  CCND1  ESR1  GPT  CTSL  PPARA  CASP7  NR1H4  SRC  STAT3  MCL1  CPT1A  MAP1LC3A  BECN1  IL2  CDH1  HMOX1  BCL2L1  STAT1  ABCC2  GLB1  CD36  MYC  MAP1LC3B  VEGFA  ACTA2  CS  NOS3  PTGS2  AGT  BAK1  MKI67  HIF1A  IL4  ALB  MAP2K1  RUNX2  MT2A  CTSK  MTTP  MYH7  LEP  FABP5  CYP27A1  RB1  JUN  TLR4  CREB1  MMP9  MAP2  H2AX  MMP2  EDN1  INS1  GJA1  MAPK8  CAT  HK2  CASP9  APP  CASP12  CTNNB1  DDIT3  CYP2D6  CYB5R3  CEBPB  FAS  HSF1  SOD1  CCNA2  ABCB11  CYP2C9  ABCB1A  CYP3A23-3A1  RAF1  TIMP2  CCND2  SPIRE1  CTSB  EIF2S1  RO60  NFE2L2  CADPS  NCF2  ACOX1  EIF4H  EGFR  ATM  CYBB  MAPT  SCD1  EEF1A1  CXCL1  MAPK3  COL3A1  MAPK1  BDNF  GATA4  SREBF1  TRIB3  CEBPA  COL1A2  MT1M  PENK  NR3C1  MIR379  FASLG  CD74  CYP1B1  VIM  GSR  RCN2  CYP7A1  MTOR  BIRC5  VDAC1  CYP2E1  MYLK  FASN  HAVCR1  MIR151  CYP1A2  IGF1  PRMT5  FABP4  ABCB1B  ATF4  KRT19  GSK3B  CSE1L  MBP  PPT1  PPM1B  IL12B  NR1H3  RAC1  BGLAP  ATP1A3  LMNA  MYH11  POLR2A  ATG5  CCL3  MPST  IGF2  REN  CCNE1  PDGFB  STAT4  SNAI1  RHEB  NEDD4  CXCL10  MAL  DYNC1H1  OPTN  GCLC  SLC39A10  ACADS  CYP2B10  CCL9  BNIP3  HSPA1B  PTPRD  MYH10  NCF1  PKM  NPPB  FOXO1  MIR31  IL17A  TYMS  CCL5  GLUL  CYP4A10  HGF  AR  CXCL2  HSPA1L  TNFAIP3  HMGCR  MIR10B  ACADL  ALDH1B1  POSTN  ELK1  LAMB1  ABCB4  ACTB  NDRG1  OCLN  ITGAM  MT1  NANOG  NQO2  TOP1  MYH6  YAP1  GEM  CDKN1B  TJP1  FN1  EIF4EBP1  IFNB1  PMAIP1  BCL2L11  GADD45A  ITGAV  KDR  PTK2B  PRKCA  ERGIC3  INTS3  PRPSAP2  HNF1BB  SUR-7  AHCY  HSP90AA1  TARDBP  EIF2AK3  HNRNPA1  CLNS1A  CAMK2A  ACACB  CTSD  GPX1  HK1  ZMPSTE24  GCLM  RGS18  COG1  NFATC1  PER3  ABCC4  CYP1A1  RNY5  LAMC2  PDIA6  ANGPTL6  APOA1  ARHGAP42A  CDF-1  ERCC1  PLPBP  SLC18A2  U2AF2  CAPN1  TPM4  LRP1  EGR1  PRL  VIPR2  MARCKS  CYP8B1  MMP1  RNY3  MBOAT7  PRKN  RAB7A  MIR29C  PRDX6  VPS45  ABCA1  PRLR  ACTA1  ATG7  RPL35  GOLGA3  CCNB1  SLC7A11  SFN  CDX2  UCHL1  PINK1  FBXO32  NOB1  PGD  FTL  TFRC  PDCD4  CAMSAP2  MTMR6  KIF20A  AQP1  ARID3A  ARPC5L  ATF2  SCFD2  DEF6  GAK  FKBP3  SCHIP1  SLC30A2  SOX2  MFF  IGSF11  G6PD  CSF2  LARP4B  ART3  DAP3  IL12RB2  ATP1A1  COL4A1  LPL  TSC22D1  SIN3A  CXCL5  ATP2C1  ME2  SCCPDH  SNU13  ACADM  ALPI  ENO2  PTGES2  RBM15  WDR26  AANAT2  PTEN  BRD8  NPPC  S100A4  EHHADH  APEX1  CKB  CSNK2B  GATAD2A  MAP2K2  NDC1  CELSR3  ITPR1  ATP6V1C1  MYEF2  SEMA4B  ACER2  PFKFB2  RGS19  EIF4G3  ETF1  MCM2  PSMD3  COX8A  EPN2  FGF1  GLG1  TAF15  PLA2G4A  YPEL1  ABCF2  BAZ1B  CSF1  DHCR7  GRIN2B  INTS6  OAS1  BID  EHD3  SLC2A2  PKIA  UPF1  IRF4  KRT16  TPP2  AP1G1  EIF4G2  AURKB  DDX6  AHSA1  IMPA1  RAB1A  SESN2  GCNT1  LAT2  CNBP  CTSA  RNY4  EDNRB  SLC12A5  EHD1  EGF  MICAL1  PPP2R1B  PZP  ABCC1  KIF1A  PDGFRB  ANO1  PTK2  RBMX  NR4A2  NUP153  PDX1  TIMP3  WNK1  FLT1  LUC7L  SEC23B  TOMM40  AOC1  MTNB  FOXO4  KRT8  PEA15  MTF1  RNY1  DNM2  TRPV2  CALR  CHUK  COLEC12  CSNK1D  MLEC  PPM1A  TGM2  ANXA2  CASP1  COX6A2  GPX4  PRKCB  TRP53  PTPRN  AKT1S1  CDK2  GNG2  SPTLC2  WIPF1  GPX2  AGXT  TESTIN  TRPV6  CYP3A11  LONP1  MPO  SYT1  ID1  MRPS6  NOTCH1  NQO1  PDP1  TXN  IL4R  MIR155  MT1H  SPAG9  SYN2  ADM  MIR34A  APOB  RPS20  APRT  DRD4  ARL4C  CCNB2  E2F1  S100G  STK39  TRPV1  SOS1  VMP1  BAIAP2  EIF3C  TLR7  IER3  SPP1  TOMM20  ATF3  HBEGF | TEX101  DUSP9  AC024587.2  HAPLN2  AC105935.1  KLK8  G023046  CATG00000027321.1  AC244502.1  AC106795.3  C20orf24  ARHGEF1  IFT52  CATG00000042513.1  AL035696.4  CATG00000066456.1  AC005740.3  LIPE-AS1  FAM167A-AS1  TALAM1  ATG5  ADIRF-AS1  AC006111.1  AC008443.2  ARHGEF9  ANKRD30BL  NKX2-2-AS1  KIAA0895L  AC104809.2  MARCKSL1  AL162258.1  CATG00000062999.1  CATG00000058631.1  SEMA3B  AC244502.3  CATG00000053562.1  AC084200.1  LINC01358  MEIG1  SRD5A3-AS1  SOCS2-AS1  CALCA  GLUD1  GUK1  AL161785.3  AC067930.1  TRIM6  RASAL2  BIG-lncRNA-582  MACROD2-AS1  EXOSC2  CATG00000004152.1  USP44  INTS6-AS1  AL592166.1  AC012594.1  G049958  TMEM114  LOC101928303  DANCR  ILDR1  FAM98A  CASC6  SLC7A7  LCN9  CATG00000011211.1  AP002387.1  CATG00000012021.1  AC126696.3  LINC01198  TMEM106B  HAGHL  NDUFA4L2  FBXO11  CTC1  DVL3  LINC01191  G053003  AP2M1  G018844  PPY  ZNF728  AC007743.1  AC092017.4  AP002748.3  LINC02092  AC008691.1  AC073941.1  AC073842.1  LINC01389  UQCC3  AL157400.3  LINC01605  ZNF529-AS1  XLOC_001120  AC079684.1  LINC01481  FARSA  AL354920.1  TRIM47  CATG00000015125.1  LACTB2-AS1  SLC4A2  CATG00000098669.1  CPA3  AP001351.1  SOX9-AS1  TMOD3  NNT-AS1  AC022196.1  CATG00000117912.1  AC004233.2  TBC1D17  ASB18  LRRC8C-DT  LINC01376  AL049651.1  CRAT  CATG00000024467.1  WFDC11  CNNM2  LINC00408  DACT3-AS1  BACH2  PPP1R26-AS1  SPRN  PARD6G  SAMMSON  ARHGEF7-AS2  ACTB  CATG00000088201.1  CATG00000068853.1  JMJD7-PLA2G4B  TMC3-AS1  RSPH6A  LINC01619  AL138689.2  LINC01425  CATG00000053260.1  HTRA2  TRIM52-AS1  MIDN  CATG00000007650.1  AL391863.1  TMEM236  AL513329.1  AC025165.3  AC092316.1  BEAN1  DLEU2  AC011442.1  SGTA  KCNN2  TNNC2  BX255925.1  CATG00000038197.1  AC068057.1  OTX1  BHLHB9  C7orf71  AC091948.1  PCDHB2  G002981  CYP11B1  KRTAP10-10  AL357054.3  HTN1  MYOZ2  AL807776.1  AL512444.1  ATG12  XKR9  LINC01588  SAMD12  AL132819.1  FABP3  FGF14-AS1  PIK3IP1-AS1  HOXC13-AS  G090757  CASC18  DNAJC17  G032205  XLOC_000223  IVNS1ABP  H2AFY  MHENCR  AC023762.1  PHF5A  ZSCAN18  SCLY  C19orf85  AC009145.2  LARP1B  G047908  LINC01821  HMGB3  XLOC_001935  AC005264.1  FAM98B  AC068418.2  XLOC_008995  FCER2  SCT  SCARF1  CATG00000114373.1  MSH5  AC004832.3  ELOA2  GPRIN2  CATG00000038258.1  ARMCX6  AC010998.3  NDUFB2-AS1  LINC00635  NBR2  DUS1L  XLOC_003243  ULBP2  MT-ND4  XLOC_003734  CTSZ  AC092723.3  TPT1-AS1  SLC35F3  G031345  AC022148.1  XLOC_001908  F10  XLOC_l2_003810  GNAS  AC015849.5  AL392172.1  LINC02028  AC005906.2  CATG00000079740.1  MCRIP2  G059429  G089183  MGMT  AX748369  EDNRA  RAB3GAP1  ZNF275  GMEB2  SPON2  CATG00000088373.1  SNHG1  HHEX  CATG00000050442.1  RFFL  G064228  KCNJ8  FAM177B  SBK2  KIAA0586  ELOVL3  G000090  CUZD1  SYNGR3  C15orf45  CCDC26  CATG00000027105.1  Z68323.1  FBXL6  PPP1CA  OR51E1  AC073529.1  LINC02217  AC139749.1  ZNRD1-AS1  AC092807.3  ISCU  DEFB119  XLOC_007190  AC113383.1  SNHG5  LOC102467216  RPL17  KIAA2013  CCDC71  CERKL  AC046136.1  MAGEA10  PRG2  K00627  PALM2  PCAT7  CATG00000008025.1  SEPT12  AC108002.2  LINC00461  TNF  AC008734.2  AC068152.1  LINC00943  CATG00000117050.1  DCTN3  VOPP1  G010459  PAK6  AC010320.3  CR769776.1  G056731  CATG00000090147.1  AC145207.2  UBXN1  RMI2  CATG00000002536.1  AL358472.5  G040233  CLDN15  GAP43  LINC02577  G029625  LINC02351  MAFA-AS1  MAPKAPK5-AS1  HSPBAP1  AC011379.2  XLOC_008343  LKAAEAR1  CATSPER2  ARHGEF7-AS1  CATG00000105091.1  LINC01920  AC010491.1  RP11-504G3.4  AC009163.6  AC103726.1  CATG00000032642.1  FAM155A-IT1  TRIO  MEGF10  CBR3-AS1  KRTAP10-5  C10orf143  LINC01431  AC090457.1  AC008627.1  PTPRG-AS1  SLC35A5  CATG00000029716.1  AC100786.1  CATG00000104066.1  KCNJ11  AP003419.2  SMAD9-IT1  MT-ND3  SUSD1  CATG00000042562.1  AC116158.1  EIF4H  SNHG16  PRKAG2-AS1  G015724  SNHG9  AC004448.2  AC114401.1  CATG00000008315.1  LINC02275  MARC1  ZNF7  AC107463.1  AC023886.1  OR1K1  OR5B3  AC009970.1  NT5C2  CD36  AL445471.2  AC012146.1  SDR39U1  MAP2K3  AC110716.2  STEAP4  RPS3  AL158825.2  MDM2  PPP1R2B  AL590644.1  LINC02005  PLK3  CD44  GPR137  HJURP  ZNF662  LINC02395  CNNM3  AC011603.3  AC053513.1  PINK1-AS  AC016757.1  AC103740.1  G049000  AKAP7  G026430  LINC00852  AL031056.2  VLDLR-AS1  SULF2  FGF1  CATG00000009357.1  SFI1  MVD  AC016629.2  CATG00000084862.1  AC159540.1  AL592429.2  AC087721.2  G047069  HHIP-AS1  CATG00000039615.1  G088994  LINC01574  AC011939.2  IFNAR1  LINC01639  LOC101927854  Z68871.1  AC097103.2  G051726  AP005328.1  AL355306.2  SALRNA2  MT-ND2  TMEM151A  ANAPC11  FAAHP1  CXCL14  GOLGA8N  MUTYH  G009112  G041405  CATG00000052478.1  LOXL1-AS1  AC018638.7  ERAL1  AP003419.3  G015277  PPP4C  NEAT1  AC004865.2  AC092153.1  AL157414.2  G068716  STAU2-AS1  NDUFA3  HELZ2  RPL35A  INTS3  CYBB  ITFG2-AS1  AP002884.1  AC010524.1  AC004982.2  SND1  AC090844.3  PPP1R16B  NAA11  AL669831.5  AC004690.2  CATG00000058838.1  SMIM36  AC091013.1  CCL4  UNC5B-AS1  AL096701.3  ELANE  AC093895.1  SLC18A1  SKIL  LINC00320  OTUB1  KIF27  CPZ  C5orf24  AC005154.4  CATG00000032673.1  CFH  PC  GADD45G  AC004870.4  CC2D1A  ZNF81  CDK10  ZNF410  SCAF1  AC092720.1  UNC5B  HLA-DOA  CATG00000116804.1  LINC00518  SNHG12 |
