# Supplementary material for: Comprehensive bioinformatics analysis and systems biology approaches to identify the interplay between COVID-19 and pericarditis
Source: Front Immunol. 2024 Feb 22;15:1264856. doi: 10.3389/fimmu.2024.1264856 (PMC10918693; doi:10.3389/fimmu.2024.1264856)
Supplement: Supplementary file 3 [file Table_2.doc]

TABLE S2 Pericarditis-related genes after filtering.

| GeneCards (n=500) | DisGeNET (n=51) | CTD (n=500) |
| --- | --- | --- |
| PRG4  TPR  TNFRSF1A  MEFV  ADA  CRP  IL6  IFNG  HLA-DRB1  TNF  IL10  VTN  C4A  AZU1  PTPN22  TRIM37  ACE  NPPB  PSTPIP1  TNNT2  CTLA4  ACTA1  STAT4  IL1B  NOD2  ITGAM  CXCL8  FCGR2A  FCGR3B  C4B  IRAK4  TNFAIP3  P2RX7  FCGR2B  DNASE1  TREX1  PDCD1  TNNI3  BLK  ETS1  IRF5  MIR1279  ALB  ENO2  PSG2  CRYAA  HLA-B  LEP  TLR4  G6PD  NAMPT  ADIPOQ  TRAF3IP2  SLC17A5  RETN  IRAK1  ENG  TLR7  CR2  MECP2  SPP1  UBE2L3  ATRIP  TNFSF4  PXK  TNIP1  JAZF1  KIAA0319L  BANK1  IGHG1  ATRIP-TREX1  SLEB3  SLEB12  SLEB13  SLEB14  SLEB15  SLEB4  SLEB5  SLEB7  SLEB8  TPT1  HRH4  ACAN  TPM2  TPM3  CFL2  STAMBP  TNNI2  ASCC1  TNNT1  TNNT3  TRIP4  NEB  SLC25A29  STAMBPL1  SELENON  TNNI1  NGRN  CINP  KBTBD13  MT-TN  PRTN3  CALB2  LACC1  MIF  HLA-DPB1  SERPINA1  MAF  IFNGR1  TTN  FAS  HLA-DPA1  CD4  ACE2  FOXP3  REN  TRPM3  FKRP  FKTN  CDAGS  TTR  MYBPC3  WBP2NL  ERAP1  PMM2  TLR5  NEU1  ADA2  SMAD4  CCR1  IL23R  P4HA2  IL12A  IRF4  HBA1  HBA2  UBAC2  KLRC4  IL12A-AS1  RNU7-1  HRAS  GNPTAB  TLR2  TBP  PTPN11  ANGPT2  L1CAM  RIT1  RYR1  ARID1A  DHCR24  NSD1  SLC26A3  RAPSN  RYR3  THSD1  DNAH9  C12orf57  LRRC56  KIF19  LOC106804612  WG  IL2  H2AC18  F3  SSB  CSF2  IL1A  CEACAM5  INE1  CCR6  CCL2  MBL2  MYD88  F2  FBN1  CD8A  IL4  PLG  IL17A  IL18  APOH  MPO  ICOSLG  IL1R1  IFNA1  CD40LG  TRIM21  ICAM1  IL1RN  HSPD1  RNPC3  TNFSF13B  SELL  HLA-DQA1  SERPINC1  THBD  VEGFA  FCGR3A  CD86  IFNB1  IL13  SELE  CSF3  STAT1  IFNA2  NAT2  CKMT2  LDHAL6B  ANKS1A  AHSP  LDHAL6A  CKMT1A  LINC00528  ATP2B1-AS1  LINC01001  NLRP3  CD19  ELANE  CD28  IFIH1  HLA-A  IL12RB1  IL5  VCAM1  CCL5  CD80  CXCL10  NLRP1  SNRNP70  MIR21  MIR142  MIR125A  MIR30A  CD79A  CD69  TF  STAT3  TP53  JAK1  PTPRC  CD40  IL2RA  ITGB2  MMP3  EDN1  VWF  CCR5  COMP  CD274  SELP  TBX21  CSF1  IL15  HLA-DQB1  CCL3  IL1RAPL2  CENPB  RO60  MIR155  MIR210  TFRC  PSME3  IL22  CYP3A4  MMP9  MMP2  TGFB1  TYK2  LCK  CALR  TLR3  BCL2  FN1  IL6R  C3  TNFRSF1B  ADAMTS13  FASLG  IFNAR1  ITGA4  PON1  C1S  CD27  CLU  ENO1  GUSB  HMGB1  INS  CD22  CD38  IDO1  ITGAX  MASP2  TLR9  TPMT  TRAF6  CTSG  IRF8  LCN2  LTF  CCR4  CCR7  FCN3  HLA-C  IFNGR2  IRF3  PADI4  TAP2  S100A9  SNRPN  LTA  CXCR5  FCGR1A  HNRNPC  IL16  KLRK1  RPLP0  CXCL9  IL15RA  MX1  SERPINB1  CXCL16  IL23A  LSM2  FAM167A  SNRPD3  MIR185  MIR223  MIR381  CASP1  PSMB8  PIK3C2A  NFKB1  PPARG  DNMT1  ZAP70  CAT  CHUK  HGF  HSP90AA1  APOE  DNMT3A  DPP4  FOS  MAPK8  NFKBIA  CASP3  CD247  CD55  CREB1  FOXO1  GATA3  HSP90AB1  IKZF1  IL2RB  NR3C1  PRLR  PTPN6  SYK  TLR8  ANXA2  CD46  CYP1A1  HPRT1  IL4R  LYN  MPL  PIK3CG  SPTAN1  TAP1  TPO  ALOX5  ANXA1  CFB  CFH  CFI  F8  IRF7  LBR  RASGRP1  C1QA  C1R  C2  C5  CD59  CSK  CYP2D6  FOXO3  FYN  IL10RA  IRF1  ISG15  LMNB1  NCF1  PLA2G2A  TNFRSF13B  ADAR  ANXA5  C1QC  CAMK4  CCR2  CISH  CX3CL1  FADD  FGF2  HAVCR2  ICOS  NFATC2  PPIA  RAG1  RBP4  SOCS1  TNFRSF13C  TNFRSF17  TNFSF13  XRCC6  AHSG  ATG5  C1QB  C5AR1  CD2  CD70  CHI3L1  CIITA  CR1  CXCL12  CXCR3  CYP2C19  GAS6  HLA-DRA  IL12B  IL21  IL21R  MBP  NCL  PRDM1  TRAF2  XRCC5  APCS  C3AR1  CEBPB  CX3CR1  DNASE1L3  FCER2  HLA-G  LTK  MAN2A1  OAS1  OCA2  OSM  PRL  SLAMF7  SNRPB  SOCS3  SRF  TNFRSF4  ATP12A  CD84  HSPA4  PROCR  CD180  CD226  COIL  CREM  DDX39B  KLRD1  RAG2  RASGRP3  RPL7  S100A8  SLAMF1  SNRPE  SRSF1  TNPO3  TRAF1  ATP4A  CCL4  CXCL13  DEK  ELF1  H1-5  HSPA1B  IL17B  LGALS8  LY9  RPLP2  SIGLEC1  SIGLEC5  SLAMF6  SSRP1  CD72  CFHR2  EVL  HLA-DMA  HLA-DMB  HLA-DRB5  IFIT3  ISG20  RCN2 | PRG4  TNFRSF1A  STAT4  CTLA4  IL10  MEFV  HLA-DPB1  HLA-DRB1  HLA-DPA1  UBAC2  HLA-B  PMM2  IL6  IL12A  MIF  PRTN3  NOD2  ERAP1  HBA2  HBA1  DNASE1  IL12A-AS1  TREX1  FAS  CCR1  KLRC4  LACC1  IL23R  FCGR2B  PTPN22  TLR4  C4A  FCGR2A  IL1B  SMUG1  IL1A  TRAF3IP2  TRIM21  CAT  PTPN2  SNRNP70  PLG  CRP  ADA  CD274  NLRP3  IL1RN  LEP  NT5E  P2RX7  MIR1279 | BAX  TNF  IFNG  IL6  MPO  IL12B  JUN  CASP3  RGS4  VEGFA  BCL2  GPT  PTGS2  VIM  CDKN1A  ACE2  EFEMP1  HMOX1  ABCG2  SPP1  MAPK3  CAT  MR1  IL10  MARCKS  RAMP1  IL1B  EDN1  SPON1  EGR1  CXCR4  ISG15  RELA  SMS  ABCC1  TP53  MAPK7  SOD2  MAPK1  IGFBP5  F3  ANGPT1  COL1A1  TGFB1  NOS2  FBLN1  PRKCZ  ALB  GSTM1  CXCL1  SLC7A11  GFAP  THBS1  DUSP1  CCL2  NFE2L2  ADAMTS9  PRC1  RRM2  CLIC1  ALDH1A1  F2  ABCC2  SCD  CXCL8  ZNF282  ABCB1  MFGE8  IL1A  CASP9  ASNS  PCNA  ERP29  CKB  BST2  NPPA  ACKR3  SOD1  PLA2G4A  EGFL6  SPARC  CTSL  TNFRSF1A  IL13RA1  IFI44  NMB  CDCA8  BDNF  NFKB1  PARK7  CCNG1  GADD45A  TIMP3  SDC1  TNNI3  TAGLN  ANXA2  GPX1  KRT8  HSPB1  NR3C1  NRG1  IFITM1  COL5A2  SLC22A7  VCAM1  PLIN2  IL2  VCL  PPP3CA  UPP1  KRT18  PLP2  PDIA4  IL12A  SGCE  DDAH2  TLR2  NQO1  CEBPB  S100A11  CTNNB1  FOS  CLGN  GDNF  SERPINE2  BTG2  TFF3  TEX261  BBC3  NGFR  PPARG  TAX1BP3  PER1  SFN  IFI44L  TOB1  AKT1  CYP3A4  DZIP1  DDIT3  PRLR  FOSL2  TMPRSS2  RNF19B  AMIGO2  JAG1  MGP  CITED2  LCN2  TJP1  ADORA2A  ITK  IFI35  PPFIBP1  HMGB2  EIF4EBP1  IGFBP3  SLPI  TXNIP  TSPO  GBP1  NRP1  VLDLR  ASL  DLC1  NR1D1  CALB2  NGF  NEDD4  G0S2  CEP41  DCK  CD44  ASS1  MCM5  TUBA1A  FGA  MELK  ATF3  TRIB3  S100A4  SUFU  A2M  PER2  MCM2  ANXA6  GINS2  PLAT  CDK5R1  CDKN1B  DRD2  GAPDH  FAM171A1  LY6E  TK1  TNFRSF12A  IFIH1  LASP1  TIMP1  MBP  DPYSL3  HSD11B2  NASP  NOC2L  TLR4  TEK  BUB3  ZHX3  GAMT  DPAGT1  ABCC6  PROC  STAT3  PARP1  BCAR3  BIK  CAMK2D  HAND2  MDM1  PPP4R2  FCGR2B  RIGI  POSTN  SERTAD1  H2AX  ESYT1  ID3  SQLE  IFI27  TNFRSF19  CAPN3  IMPDH1  NETO2  RHOC  FGF7  PTPN18  CTSH  ARHGDIB  FGF5  PTCH1  SFRP1  IGF1  MT1A  LY96  ZWINT  TES  MVP  MAPK14  TNFSF10  ADM  PPP1R1B  COX14  COL11A2  CYB561  MAP3K12  UQCR11  STAT5A  GNAI2  CYP1A2  FMOD  NBL1  LUM  ABCC3  ENO3  B3GNT9  AHNAK  MSH2  NDC80  SLC47A1  ENO1  SLC43A3  UGT1A1  MAFF  MMP10  PLK3  PRPH  RAN  ADIPOQ  BID  SERPINE1  IL17A  TYMS  LRRN3  PXYLP1  DAB2  S100A10  ID2  ABCB1A  HGF  MX1  SLC16A3  BCAS4  GADD45B  DES  IFIT3  TNFAIP3  AKAP12  HINT1  DTX4  PIK3C2B  KIF2C  CCN1  CCN4  UNG  IL18  MEX3B  IL6R  NAT2  COL18A1  TGFB2  CD9  IL1RN  OCLN  TPM3  CD14  MMP3  SEC31A  ANXA4  SGK1  ADGRL2  INVS  MPZL1  ANKRD13D  XRCC1  CD55  TAP2  SPARCL1  TCF12  DEPDC1  MYO1E  TRP53  EFHD2  PARD3  ZFHX4  DNMT1  ERBB2  CDC25A  CFB  MET  E2F8  GPM6B  NFATC3  SERINC2  DNAJC27  FOSL1  HPGD  MYADM  NFE2L3  TFE3  CAV1  CXCL2  DDIT4  ADIRF  BAZ1A  TLN2  ACSS1  CSTA  ETHE1  FOXN3  BUB1B  HSPB8  CD34  ARG2  COLEC12  ORM1  SRXN1  EMP3  ERBB4  RRAS  TAC1  HP  PISD  FAM234B  RGS13  BIRC3  NDUFS4  PCOLCE  COL3A1  EEF2  HMGB1  CDKN3  LMNB1  PDPN  TAFA5  DGCR6  FST  LIMD1  SOCS7  STAU1  PPP1CA  RANBP1  CDKN1C  EZR  MTHFR  NID2  UCK2  DGKA  FNBP1  HSPD1  CREM  MMP9  PPT2  TFCP2  GSDME  NEK6  PPP1CB  IRF6  COL1A2  CKAP4  KCTD12  CBR1  CYP27A1  DRD1  UBE2C  CXCL12  GRAMD4  TUBD1  PLP1  MTR  NECTIN2  HDAC1  IRF7  COX5A  MAPK13  SOD3  CRHBP  DNAH2  RHBDD1  SELENOP  UPRT  C6  CA12  GPC3  IFITM2  SAMHD1  TMSB4X  ANTXR1  DIAPH3  E2F3  MFAP4  RGS1  TGFBR3  OIP5-AS1  ODC1  FANCI  PKP2  DHFR  PRL  COL8A1  CREB1  FSHB  MXRA5  PNMA2  CFI  METTL7A  MAP2K2  MMP2  FSCN1  OXCT1  KRT19  MCM6  OAT  GNG10  KLHL41  MAD2L2  NKAIN1  PMF1  SCN2B  ST6GALNAC3  RBPMS  RIPK2  CAVIN2  NFKBIE  ORC6  RASGRP1  RRBP1  ACTC1  FOSB  NID1  OLFML3  CYP1B1  GSTA2  FOXRED2  PATZ1  IRF9  PTGR1  RC3H1  REPS1  YIF1A  APOC1  FZD2  ANKRD45  AP3S1  LMCD1  ERH  PTMA  RFC3  RPP25  CASD1 |
