# Supplementary material for: Comprehensive bioinformatics analysis and systems biology approaches to identify the interplay between COVID-19 and pericarditis
Source: Front Immunol. 2024 Feb 22;15:1264856. doi: 10.3389/fimmu.2024.1264856 (PMC10918693; doi:10.3389/fimmu.2024.1264856)
Supplement: Supplementary file 4 [file Table_3.doc]

TABLE S3 Common genes of COVID-19 and pericarditis (n=313).

| ATG5  FCER2  PPP1CA  TNF  PLK3  CD44  IFNAR1  CCL4  ELANE  CFH  ACE2  TLR7  TMPRSS2  IL6  ACE  CRP  CXCL10  CXCL8  IL1B  CCL2  IL10  NLRP3  F3  NRP1  TNNI3  IL2RA  DPP4  F2  IL2  IFNB1  IL4  HLA-A  CTSL  CCR6  CCL3  TYK2  REN  HLA-C  IFNA2  TLR4  VWF  IFNA1  IRF3  IL17A  ALB  CD8A  NPPB  IL22  IFNG  PLG  NFKB1  GPT  CSF2  IL18  OAS1  TNFRSF1A  TNFRSF1B  CD4  INS  NFKBIA  FCGR2A  RELA  TLR3 | FCGR3A  APOE  CSF3  STAT1  SERPINE1  CCR5  ADAMTS13  FCGR3B  MBL2  S100A9  HLA-B  HMGB1  S100A8  HMOX1  JAK1  MIR21  STAT3  HLA-DRB1  ADM  CD274  IFIH1  MIR155  SERPINA1  G6PD  PDCD1  SIGLEC5  ANGPT2  TGFB1  CD14  IL6R  SERPINC1  ISG15  TLR2  SELP  P2RX7  IL13  IRF7  HAVCR2  VEGFA  BDNF  FAS  CCL5  SIGLEC1  CD28  FGA  IGF1  IL1A  TNNT2  CHI3L1  NFE2L2  TLR9  CTLA4  THBD  MPO  CCR2  CCR1  TLR8  NEU1  HLA-G  CXCL1  ANXA2  TTR  ALOX5 | MTHFR  EDN1  C5AR1  IRF9  IDO1  IFITM1  SOCS1  LCN2  SDC1  MYD88  C5  IFI44  MX1  RIGI  IL15  CXCL2  IL2RB  STAT5A  FOXP3  CD80  CD86  PROCR  MIR142  CXCL9  NR3C1  GPX1  DNMT1  AKT1  IFNGR1  TES  LMNB1  GAS6  SELL  ICAM1  CXCL16  NEDD4  ICOSLG  TEK  TF  ANXA1  SOD2  PLA2G2A  GSTM1  S100A4  S100A10  MIR30A  ITK  CAT  PSME3  ADIPOQ  ADAR  PON1  PLAT  HBA1  F8  CD19  ERBB2  IL1RN  ABCB1  C3  CYP3A4  LTF  MAPK1 | CASP1  OCA2  CASP3  JUN  MEFV  IL23A  PARP1  ANGPT1  CD38  CDKN3  CYP2D6  MET  MMP9  PIK3CG  PPARG  PROC  CD40LG  CSF1  CFI  ISG20  ITGAM  LEP  NOS2  MAPK8  PTGS2  PTPRC  CALR  COL11A2  FN1  GFAP  CXCR3  IL5  ITGAX  NT5E  CXCR4  BCAR3  TSPO  CAV1  CEACAM5  DDIT3  DNASE1  HP  IL1R1  IRF1  ITGB2  KRT18  LY6E  ABCC1  PPIA  MAPK3  SELE  SELENOP  SOD1  TRIM21  TLR5  SGCE  TP53  BAX  PCNA  BCL2  CDKN1A  BBC3 | FOS  COL1A1  ABCC2  CYP27A1  CREB1  H2AX  MMP2  CASP9  CTNNB1  CEBPB  ABCB1A  RO60  COL3A1  TRIB3  COL1A2  FASLG  CYP1B1  VIM  RCN2  CYP1A2  KRT19  MBP  IL12B  STAT4  HSPA1B  NCF1  FOXO1  TYMS  HGF  TNFAIP3  POSTN  OCLN  CDKN1B  TJP1  EIF4EBP1  GADD45A  HSP90AA1  CYP1A1  EGR1  PRL  MARCKS  PRLR  ACTA1  SLC7A11  SFN  TFRC  ENO2  CKB  MAP2K2  MCM2  PLA2G4A  BID  IRF4  TIMP3  KRT8  CHUK  COLEC12  TRP53  NQO1  IL4R  SPP1  ATF3 |
| --- | --- | --- | --- | --- |
